# Quantitative V gene–targeted T cell receptor sequencing as a biomarker in type 1 diabetes

**DOI:** 10.1172/jci.insight.186004

**Published:** 2025-12-18

**Authors:** Laurie G. Landry, Kristen L. Wells, Amanda M. Anderson, Kristen R. Miller, Kenneth L. Jones, Aaron W. Michels, Maki Nakayama

**Affiliations:** 1 Barbara Davis Center for Childhood Diabetes, and; 2 Department of Pediatrics, University of Colorado School of Medicine, Aurora, Colorado, USA.; 3 Bioinformatic Solutions, LLC, Sheridan, Wyoming, USA.; 4 Department of Immunology and Microbiology, University of Colorado School of Medicine, Aurora, Colorado, USA.

**Keywords:** Autoimmunity, Immunology, Autoimmune diseases, T cell receptor

## Abstract

Developing biomarkers to quantitatively monitor disease-specific T cell activity is crucial for assessing type 1 diabetes (T1D) progression and evaluating immunotherapies. This study presents an approach using V gene–targeted sequencing to quantify T cell receptor (TCR) clonotypes as biomarkers for pathogenic T cells in T1D. We identified “public” TCR clonotypes shared among multiple nonobese diabetic (NOD) mice and human organ donors, with a subset expressed exclusively by islet-antigen-reactive T cells in those with T1D. Employing V gene–targeted sequencing of only TCRs containing TRAV16/16D allowed quantitative detection of the public islet-antigen-reactive TCR clonotypes in peripheral blood of NOD mice. Frequencies of these public TCR clonotypes distinguished prediabetic NOD mice from those protected from diabetes. In human islets, public TCR clonotypes identical to preproinsulin-specific clones were exclusively found in T1D donors. This quantifiable TCR sequencing approach uncovered public, disease-specific clonotypes in T1D, providing biomarker candidates to monitor pathogenic T cell frequencies in blood for assessing disease activity and therapeutic response.

## Abstract 2

This study provides a proof-of-concept to utilize immune-receptor sequences to monitor antigen-specific T cells in the blood, predicting progression of autoimmune diseases.

## Introduction

Development of biomarkers associated with disease activity and progression is imperative for the clinical management of patients with type 1 diabetes (T1D) (1). We are able to predict the development of T1D in high-risk individuals by detecting autoantibodies against pancreatic β cell antigens (2, 3). However, there remains a crucial need for biomarkers that mirror the presence of disease-specific T cells in the pancreas, allowing the assessment of disease activity in individuals developing T1D as well as those undergoing immunotherapies to impede immune-mediated β cell destruction (2, 4, 5). Quantitative detection of islet-antigen-specific T cells in peripheral blood poses a challenge due to their rarity (6). Moreover, islet-specific T cells are often found in the blood of nondiabetic healthy individuals, leading to limited differentiation between individuals with various stages of T1D (7, 8).

There are subsets of T cells in the pancreas of patients with T1D that are distinct from those in nondiabetic individuals (8, 9). Although islet-specific T cells may be present in the pancreas regardless of disease status (8–10), the recognition of antigens by T cell receptors (TCRs) of T1D donors is significantly more intense compared with nondiabetic donors (9). Consequently, TCR clonotype information can delineate characteristics specific to T cells involved in T1D pathogenesis occurring only in the pancreas of patients with T1D (4, 11). Recent advancements in sequencing technologies offer opportunities to develop T cell biomarkers utilizing TCR sequences. Numerous studies demonstrate that diversity and clonality of TCR repertoires correlate with prognosis and therapy efficacies in patients with cancer and transplant recipients (12–16). Hundreds of thousands of sequence reads may provide sufficient analysis depth to detect extremely rare clonotypes, which will be advantageous to overcome difficulties in developing T cell biomarkers for autoimmune diseases.

Hence, we hypothesized that clonotype-based biomarkers, specifically enumerating TCR clonotypes expressed by T1D-specific T cells in peripheral blood samples, may serve as surrogate markers for T cells that can recapitulate pathogenic T cell activity in the pancreas of individuals developing T1D (4, 11). TCR clonotypes assessed in blood samples should satisfy 3 essential conditions to be used as biomarker: (a) sufficient abundance for quantitative enumeration (abundance), (b) expression by T cells across multiple individuals (publicity), and (c) differential detection only in individuals developing or having developed T1D but not in nondiabetic individuals (disease specificity). To this end, we utilized nonobese diabetic (NOD) mice, which spontaneously develop autoimmune diabetes, alongside genetically manipulated mice protected from T1D development, to simulate whether we can identify “public” and “disease-specific” TCR clonotypes shared exclusively among T1D-susceptible animals and whether such clonotypes are “abundant” enough to be detected in the peripheral blood of animals developing T1D. Given evidence that TCRs recognizing the same peptide-MHC complex often utilize the same TCR variable gene (V gene) (17–20), a segment consisting of TCR α and β chains, we endeavored to enrich a cell population containing T1D-specific T cells by amplifying only TCRs containing the V gene that is preferentially used by T1D-specific TCR clonotypes. This approach facilitated quantitative evaluation of the presence of T1D-specific TCR clonotypes in blood samples. Furthermore, to assess the applicability of this approach to human T1D, we validated the sharing and disease association of TCR clonotypes detected in islet samples from organ donors with or without T1D. Here we provide evidence of TCR clonotypes shared between individuals, including those expressed by islet-antigen-specific T cells, which may reflect disease association in the pancreas of organ donors with T1D.

## Results

### TCR repertoires in pancreatic islets of prediabetic NOD mice.

**Figure 1:**
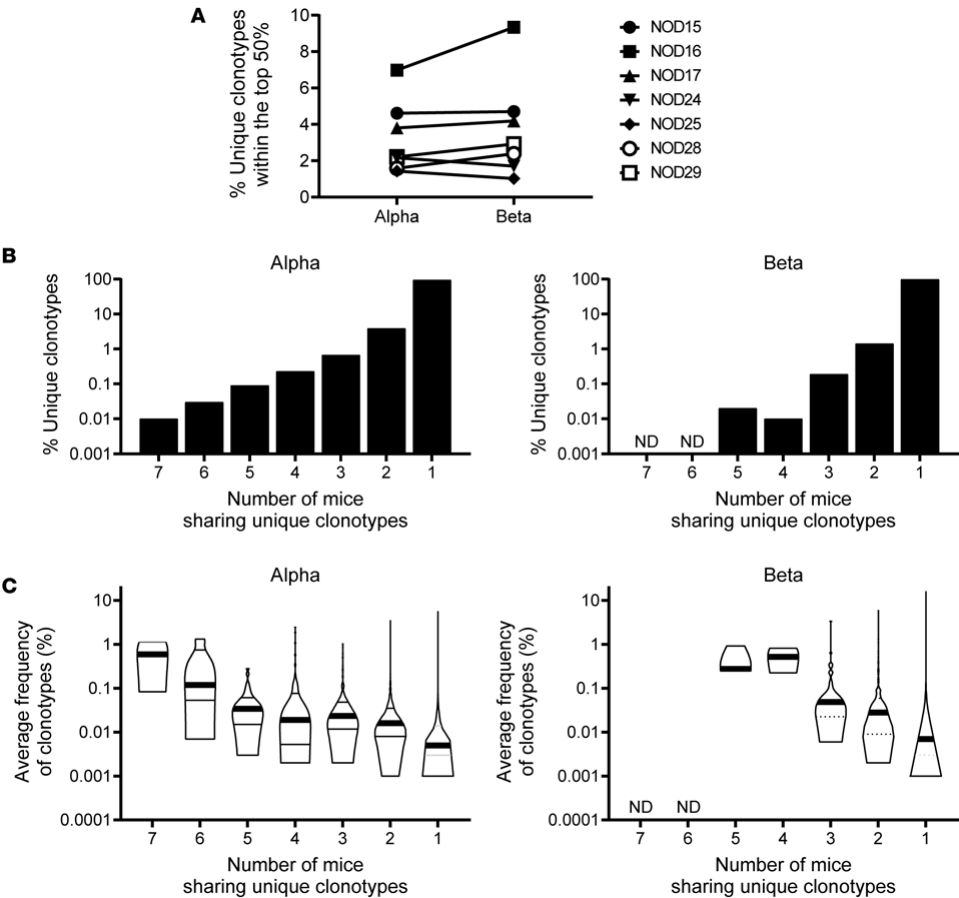
Landscape of TCR repertoires in the islets of NOD mice. TCR α and β chain clonotypes expressed by T cells in the islets of 7 prediabetic adult NOD mice were determined and analyzed for frequencies and sharing between individual mice. (**A**) Percentages of unique α and β clonotypes that account for top 50% ranked sequence reads in each mouse are plotted. Smaller percentages imply that TCR repertoires are more deviated toward particular clonotypes. (**B**) Percentages of TCR α (left panel) and β clonotypes (right panel) detected from different numbers of mice are plotted. Over 95% of clonotypes were detected from only a single mouse. (**C**) Frequencies of TCR α (left panel) and β clonotypes (right panel) detected from different numbers of mice are shown in violin plots. There were no β clonotypes that were detected from 6 or 7 mice. There was a significant difference between number of mice sharing a clonotype and average frequency of clonotypes (overall *F* test *P* < 0.0001 for both α and β clonotypes assessed by 1-way ANOVA with Tukey’s honestly significant difference [HSD] test).

Public TCR clonotypes, which are commonly expressed by disease-associated T cells, are expected to be present in targeted organs. Therefore, we first analyzed TCR repertoires in the islets of NOD mice to identify such public TCR clonotypes. We isolated islets from 7 female prediabetic adult mice aged 7 to 9 weeks, known to exhibit significant T cell infiltration and sequenced TCR α and β chain genes. We acquired a total of 2.7 × 10^5^ TCR α and 2.3 × 10^5^ TCR β productive sequence reads (i.e., in-frame and no stop codon in CDR3), identifying 22,994 unique α and 16,795 unique β clonotypes (Supplemental Table 3; supplemental material available online with this article; https://doi.org/10.1172/jci.insight.186004DS1). Proportions of frequent TCR clonotypes that account for 50% of a whole TCR repertoire were only a few percent of all unique clonotypes detected (Figure 1A and Supplemental Figure 1), indicating clonal expansions or accumulations of T cells in the islets.

### Identification of public TCR clonotypes in the islets of NOD mice.

We next set out to determine whether specific TCR clonotypes were shared among individual mice, potentially serving as biomarkers for T1D development. Comparing the TCR α and β repertoires between any 2 mice showed considerable variance, with only a few percent of unique clonotypes shared (Supplemental Figure 2 and 3). Notably, 95% of α and 98% of β were unique to individual mice, and only a small portion of clonotypes were shared by 2 or more mice (Figure 1B). However, clonotypes shared by multiple mice were more prevalent than those that were detected in only 1 mouse (Figure 1C, *P* <0.0001 in both α and β clonotypes), suggesting that T cells expressing shared “public” clonotypes are more abundant in islets than those expressing nonshared ones. Although the majority of TCR clonotypes are private (i.e., not shared between individual mice), several commonly detected clonotypes were prevalent and could be used as TCR biomarkers. Specifically, we found 10 TCR α clonotypes that were shared by 6 or 7 out of all the 7 mice analyzed (Table 1). Two sequences, TRAV11-CVVGDRGSALGRLHF-TRAJ18 and TRAV11-CVVVDRGSALGRLHF-TRAJ18, are typically expressed by natural killer T (NKT) cells (21) and accounted for 1.2% of the total sequences. Likewise, mouse mucosal-associated invariant T (MAIT) cells usually express an α chain containing TRAV1, and thus TRAV1-CAVRMPNYNVLYF-TRAJ21 may be expressed by MAIT, although the J gene usage is atypical (22). Two other public clonotypes, both with identical junction sequence (CAMRDSGGSNAKLTF) along with V-α gene segment TRAV16 or TRAV16D and J-α gene segment TRAJ42, were very prevalent, accounting for an average of 1.3% and 1.1% in the islets of each animal (Table 1). Strikingly, these clonotype sequences are identical to an α chain used by the diabetogenic CD8^+^ T cell clone NY8.3, previously established from NOD islets by Santamaria et al. and known to be reactive to a peptide derived from islet-specific glucose-6-phosphatase catalytic subunit–related protein (IGRP) (17, 23–25). Three other clonotypes comprised of TRAV5D-4 and TRAJ53, known to be preferentially used by autoreactive T cells recognizing an insulin B chain peptide 9–23, were also identified as public clonotypes. To verify whether T cells expressing these TCR α clonotypes are consistently present in NOD islets, we analyzed TCR sequences in islet samples of 2 additional cohorts from independent NOD litters (Supplemental Table 3). The frequencies of shared clonotypes showed a pattern similar to that of the original cohort (Supplemental Figure 4), with the great majority of clonotypes being private. Nevertheless, most clonotypes initially identified as public in the original cohort were again detected in all, or in 3 out of 4, mice in each of the additional cohorts (Table 1). Notably, the clonotype expressed by the NY8.3 clone was detected across all 8 animals at high frequencies.

These findings suggest that several TCR α chain clonotypes, including those expressed by islet-antigen-specific T cells, were commonly detected in the islets and may serve as biomarkers for quantifying antigen-specific T cells in peripheral blood.

### The public TCR clonotype is specific to an islet-derived peptide.

**Figure 2:**
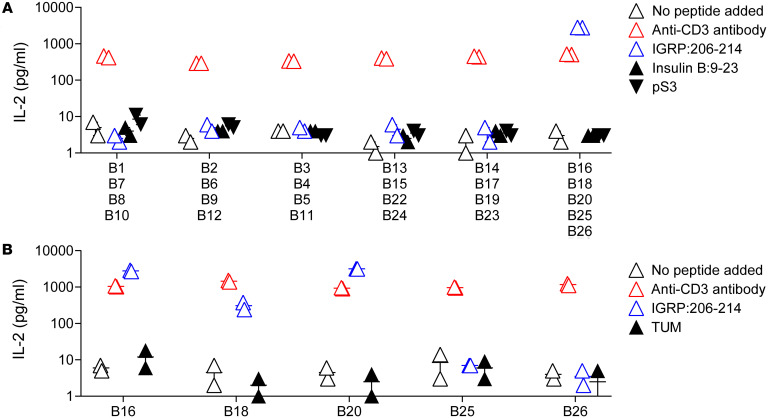
Reactivity to islet antigens by TCRs composed of public NY8.3 α. (**A**) TCR transductants expressing public NY8.3 α along with a pool of 4 or 5 frequent β clonotypes were tested for the response to IGRP peptide 206–214 (blue triangles), insulin B chain peptide 9–23 (black triangles), and a pS3 mimotope of hybrid insulin–chromogranin A peptide (black inverted triangles) in the presence of NOD spleen cells. (**B**) TCR transductants expressing public NY8.3 along with each β clonotype (B16, B18, B20, B25, or B26) were tested for the response to IGRP peptide 206–214 (blue triangles) and a control TUM peptide (black triangles) in the presence of NOD spleen cells. Cultures without peptides (white triangles) as well as those with anti-CD3 antibody (red triangles) were included as positive and negative controls in each assay. The amounts of IL-2 in culture supernatant were measured by ELISA. Representative results performed in duplicates from 3 repeated experiments are shown.

Among the defined public islet-specific TCR clonotypes (Table 1), those composed of TRAV16/16D and TRAJ42 along with the junction sequence CAMRDSGGSNAKLTF (termed “public NY8.3” hereafter) were more abundant in the islets than the other public clonotypes and may be appropriate for quantitative analysis. Therefore, we chose these NY8.3 clonotypes to pursue quantifiable TCR biomarker development. To determine antigen specificity of the public NY8.3 clonotypes, we examined the β chain clonotypes likely paired with this α required for TCR function. Assuming that frequency of paired β clonotypes would correlate with that of their α mate, we selected the top 25 frequent β chains as candidate parings (Supplemental Table 4). We tested T cell transductants expressing these candidate TCR β clonotypes with the public NY8.3 α for the response to 3 major islet-associated antigens, and one group containing B16, B18, B20, B25, and B26 responded to IGRP peptide 206–214 (IGRP:206–214) (Figure 2A). Further testing confirmed 3 of these β chains (B16, B18, and B20) as reactive to the IGRP:206–214 peptide (Figure 2B). These β clonotypes share the common V gene segment TRBV13-3 and J gene segment TRBJ2-4, but have distinct junction sequences (Supplemental Table 4). These results are aligned with previous studies by Santamaria and colleagues, demonstrating that the public NY8.3 α can pair with multiple β chain clonotypes to recognize IGRP:206–214 (17, 23–25). Thus, the public NY8.3 α TCR clonotypes are confirmed as specific for an islet antigen and may be useful for enumerating T cells specific for this peptide.

### TCR repertoires shared between islets and peripheral immune organs.

**Figure 3:**
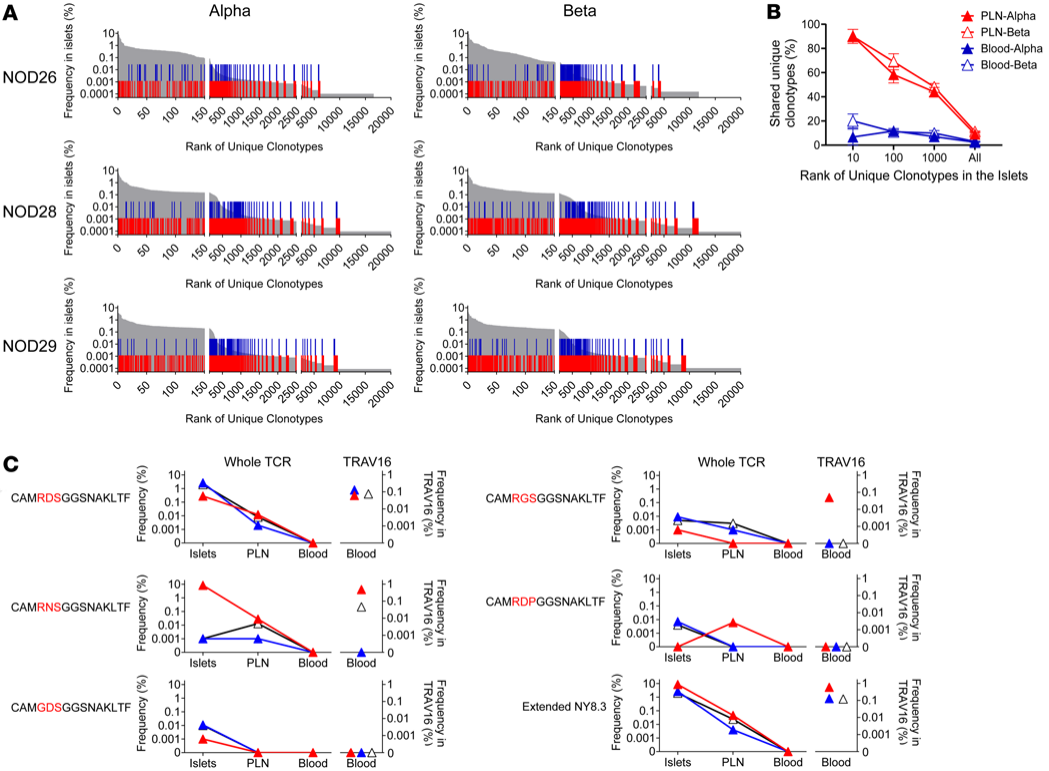
Comparison of TCR repertoires between islets, pancreatic lymph nodes, and peripheral blood. TCR α and β clonotypes in the islets, pancreatic lymph nodes (PLNs), and peripheral blood of 3 prediabetic adult NOD mice were determined and analyzed for frequencies and sharing between the tissues. (**A**) Individual unique clonotypes are aligned in the order of prevalence in the islets (*x* axis), and frequencies of clonotypes in the islets were plotted in gray (*y* axis). Clonotypes that were detected in PLNs and blood samples of the same mouse are marked with red and blue bars, respectively. (**B**) The association between the frequency of unique clonotypes ranked within 10, 100, and 1,000 in the islets as well as all clonotypes detected in the islets and the percentage of those clonotypes in PLNs (red) and blood samples (blue) are shown. Filled and open triangles represent α and β, respectively. A linear mixed model was fit to the percentage of shared unique clonotypes by rank, accounting for correlation within each mouse. An interaction between categorical rank by group (PLNs vs. blood) was included and significant differences between PLNs and blood samples at each rank were assessed using least square means (Supplemental Table 6). (**C**) Frequencies of public NY8.3 (top panels), sub-public NY8.3 (panels in between), and all extended NY8.3 clonotypes (bottom panels) in the islets, PLNs, and blood samples that were determined by whole TCR sequencing are shown in left panels. Right panels show frequencies of public, sub-public, and extended NY8.3 clonotypes in blood samples that were determined by TRAV16-targeted sequencing. Each color designates each mouse studied (red NOD26, blue NOD28, and white NOD29).

Public TCR clonotypes that are expressed by tissue-specific T cells were identified in pancreatic islets. A key question is whether such public TCR clonotypes detected in a targeted organ also appear in the peripheral blood of the same individuals, potentially serving as biomarkers. To address this, we assessed the prevalence of TCR repertoires in both the islets and peripheral immune organs, including pancreatic lymph nodes (PLNs) and blood. We analyzed millions of TCR sequence reads from PLNs and blood samples of three 8- to 9-week-old female NOD mice to compare them with the repertoires in the islets of each mouse (Supplemental Table 5). The proportions of islet-derived TCR clonotypes were higher in PLNs than in blood, with approximately 10% of TCR clonotypes in PLNs shared with those detected in the islets, compared with only 2%–3% in the blood (Figure 3A). Notably, clonotypes frequent in the islets were more prevalent in PLNs and blood samples than those that were infrequent in the islets (Figure 3B and Supplemental Table 6).

These results indicate a dilution in the frequency of islet TCR clonotypes when tracing into the peripheral immune organs from PLN to blood, suggesting that clonotypes frequent in the target organ (i.e., islets) are more detectable in the blood than less frequent ones.

### Tracing public TCR clonotypes in the blood using V gene–specific sequencing.

We investigated whether the public NY8.3 α clonotypes are detectable in the peripheral immune organs of the 3 NOD mice analyzed above. Despite a significant presence of NY8.3 in the islets, these clonotypes were undetectable in over 8 million whole TCR α chain sequence reads from the blood samples (Figure 3C, top panel). We hypothesized that selectively sequencing TCRs containing TRAV16 would enrich for the public clonotypes, making them detectable. To test this hypothesis, we exclusively amplified TCR α chain genes containing TRAV16 and TRAV16D using a primer specific to a conserved sequence in TRAV16 and TRAV16D with another primer targeting the TCR α constant region. Using this approach, public NY8.3 was detected in all 3 NOD blood samples, representing approximately 0.1% of the TRAV16 subpopulation in each mouse (Figure 3C, top panel). Furthermore, analyzing “sub-public” NY8.3 clonotypes composed of TRAV16/16D and TRAJ42 with similar junction sequences to public NY8.3, found in at least 4 of the 7 mice analyzed in the original cohort and confirmed to be consistently detected in the additional 2 cohorts (Table 2), revealed that these clonotypes were captured in the blood when using TRAV16/16D-specific sequencing (Figure 3C). The addition of these sub-public NY8.3 clonotypes likely enhances the overall detection of islet-derived TCR clonotypes in blood samples, especially in animals with a low number of public NY8.3 but a higher number of sub-public variants.

In summary, V gene–targeted sequencing significantly improves our ability to detect public TCR clonotypes in the periphery, offering a promising approach for monitoring TCR biomarkers in blood.

### Disease association of quantifiable public TCR clonotypes in the blood.

**Figure 4:**
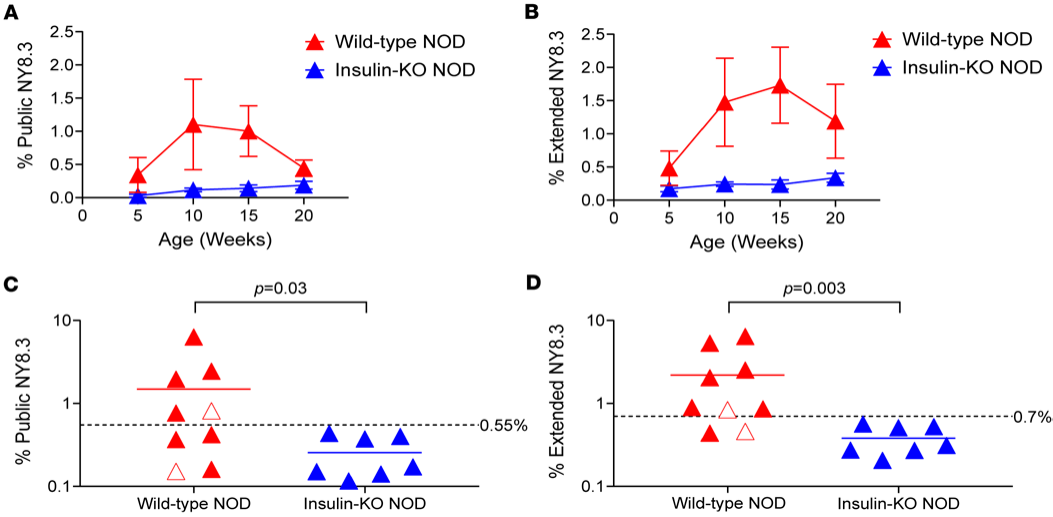
Longitudinal analysis of NY8.3 α clonotypes in the peripheral blood. TCR α clonotypes containing TRAV16 in blood samples of wild-type NOD (*n* = 9) and insulin-KO NOD mice (*n* = 7) were determined using TRAV16-targeted sequencing. The average frequency of public NY8.3 (**A**) and extended NY8.3 (**B**) of wild-type NOD (red) and insulin-KO NOD (blue) is plotted for blood samples taken from 5 to 20 weeks of age. Blood samples taken at the diabetic end sampling point for each mouse are included in the next age set. The highest values of individual mice during the study period are plotted for public NY8.3 (**C**) and extended NY8.3 (**D**). The dashed line is the cutoff value determined by the 99th percentile of highest values in insulin-KO mice. White triangle symbols represent data from NOD mice that did not develop diabetes during the study period. Fisher’s exact tests were conducted to test for differences in >0.55% public NY8.3 (**C**) (*P* = 0.03) and >0.7% extended NY8.3 (**D**) (*P* = 0.003) between wild-type NOD and insulin-KO NOD.

We further explored whether the presence of public NY8.3 clonotypes in the peripheral blood correlates quantitatively with diabetes development in NOD mice. Using TRAV16-targeted sequencing, we periodically measured the frequency of public and sub-public NY8.3 α clonotypes (termed “extended NY8.3” hereafter) in the peripheral blood of NOD mice over several weeks. Seven of 9 NOD mice developed diabetes, whereas the remaining 2 mice did not develop diabetes by 20 weeks of age. In comparison, 7 NOD mice lacking native insulin genes (insulin-KO mice hereafter), which are protected from diabetes (26), were also included in the study (Supplemental Table 7). Of note, it is known that autoreactivity to IGRP is abrogated in mice deprived of autoimmunity to insulin (27); thus, IGRP-reactive T cells including those expressing public NY8.3 are expected to stay infrequent. Although not statistically significant, there was a tendency for increased public NY8.3 clonotypes in the peripheral blood of NOD mice by 10 weeks of age, prior to diabetes onset, continuing through the 15-week time point (Figure 4A and Supplemental Figure 5A). In contrast, public NY8.3 levels remained low in all insulin-KO mice throughout the 20-week period (Figure 4A and Supplemental Figure 5B). When considering extended NY8.3 clonotypes, their elevation in the blood of NOD mice became more significant (Figure 4B and Supplemental Figure 5, C and D), allowing for clearer differentiation between NOD mice and insulin-KO mice through peak value analysis that compares the highest levels of public and extended NY8.3 clonotypes during the study period (Figure 4, C and D). Using the 99th percentile of peak NY8.3 values in insulin-KO mice as a cutoff for positivity, 5 of 9 NOD mice were considered positive (Figure 4C, *P* = 0.03). Including sub-public NY8.3 clonotypes identified 2 additional NOD mice as positive, with none from the insulin-KO group (Figure 4D, *P* = 0.003). Of note, the 2 NOD mice that remained nondiabetic had relatively lower peak values compared with those that developed diabetes (Figure 4, C and D). These findings were further validated in additional cohorts, in which extended NY8.3 clonotype frequencies were elevated in 10 of 12 wild-type NOD mice, while all 8 insulin-KO mice remained negative (Supplemental Figure 6, peak value comparison: *P* = 0.001 [public NY8.3], *P* < 0.001 [extended NY8.3]; and Supplemental Table 7). It is worth mentioning that the 2 NOD mice without elevated NY8.3 clonotypes remained normoglycemic throughout the study. Overall, V gene–targeted sequencing effectively detected elevated levels of public and extended NY8.3 clonotypes in the peripheral blood of NOD mice prior to onset of diabetes, while nondiabetic control mice maintained low levels throughout the study period. The inclusion of sub-public NY8.3 clonotypes further accentuated the differentiation between wild-type NOD and control mice.

### Presence of disease-associated public TCR clonotypes in human pancreatic islets.

**Figure 5:**
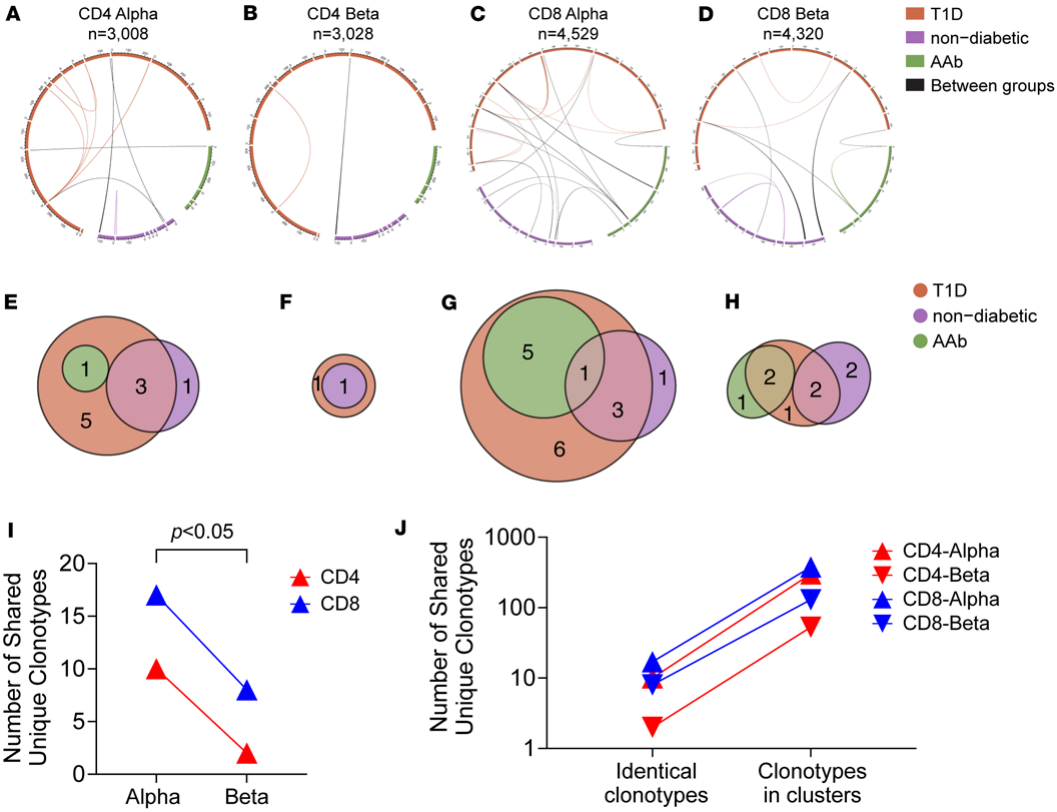
TCR sequences shared between pancreatic tissues of organ donors with or without different stages of T1D. TCR α and β chain sequences expressed by CD4^+^ and CD8^+^ T cells in pancreatic tissues of organ donors were determined and analyzed for sharing between individual donors. (**A**–**D**) TCR clonotypes of T1D, autoantibody-positive prediabetic (AAb), and nondiabetic organ donors are shown in orange, green, and violet, and those shared between the different groups are connected by black lines. Thickness of lines represents frequency of TCR clonotypes in each donor. Shared TCR α and β chain clonotypes expressed by CD4^+^ T cells are shown in panels **A** and **B**, respectively, and those by CD8^+^ T cells are shown in panels **C** and **D**. (**E**–**H**) The numbers of CD4α (**E**), CD4β (**F**), CD8α (**G**), and CD8β (**H**) chain clonotypes shared between the groups are shown in Venn diagrams with the same color coordination used in panels **A**–**D**. (**I**) The numbers of α and β clonotypes expressed by CD4^+^ (red) and CD8^+^ (blue) T cells were compared by a paired, 2-tailed *t* test (*P* = 0.037). (**J**) The numbers of clonotypes that are exactly identical between donors and those that are clustered are shown (CD4α: red triangles, CD4β: red inverse triangles, CD8α: blue triangles, and CD8β: blue inverse triangles).

Identification of public clonotypes is crucial for the TCR V gene–targeted sequencing assay to enumerate such clonotypes in peripheral blood. Building on evidence from NOD mice that we identified public TCR clonotypes from T cells in the islets, we investigated whether public clonotypes could be found in the pancreas of human organ donors having T1D. After excluding T cells expressing TCRs likely derived from MAIT cells (i.e., TRAV1-2 with TRAJ33, TRAJ12, or TRAJ20) or NKT cells (i.e., TRAV10 with TRAJ18), we analyzed TCR α and β chain clonotypes from 3,409 CD4^+^ and 4,909 CD8^+^ T cells in the islets of 27 organ donors with and without T1D (Supplemental Tables 8 and 9). Among 10,371 unique clonotypes identified, the majority were detected only from a single donor, but 47 clonotypes were shared between individuals (Figure 5, A–H, and Supplemental Table 10). Similar to observations in NOD mice, α chains shared more identical clonotypes than β chains (*P* = 0.037) (Figure 5I). Although CD8^+^ T cells had more shared clonotypes than CD4^+^ T cells, the frequencies of shared clonotypes did not significantly differ between CD4^+^ and CD8^+^ T cells based on a bootstrapping approach (Supplemental Table 11). A portion of shared clonotypes were present in both T1D and non-T1D donors, suggesting they may not be disease associated. However, it is notable that 4 of the 47 shared clonotypes were identical to α clonotypes expressed by preproinsulin-specific CD8^+^ T cells, all detected only in T1D or prediabetic (i.e., autoantibody-positive) donors (Supplemental Table 10). These results demonstrate the presence of public TCR clonotypes in pancreatic islets shared between multiple human donors, with a subset of clonotypes shared only among T1D donors being identical to islet-antigen-specific clonotypes.

### Clustering TCR clonotypes expressed by T cells in human pancreatic islets.

Given the diversity of TCR repertoires in the pancreas and the limited frequencies of public clonotypes shared between individuals, it is crucial to increase disease-associated public clonotypes for enumeration in TCR biomarker assays. Indeed, inclusion of extended NY8.3 clonotypes improved the identification of mice developing T1D (Figure 4). We therefore aimed to identify clonotypes having similar motifs using the TCRdist clustering tool (28). The algorithm identified 137 CD4α, 27 CD4β, 167 CD8α, and 61 CD8β clusters (Supplemental Figure 7), resulting in an increase in potentially public clonotypes contained in clusters (Figure 5J). Among these clusters, 2 CD4α, 1 CD4β, 14 CD8α, and 5 CD8β clusters contained TCR clonotypes expressed by preproinsulin-specific T cells. Clusters containing preproinsulin-specific clonotypes were predominantly composed of those from only T1D or autoantibody-positive donors, a trend that was found to be significant (Supplemental Table 12, *P* = 0.028 by χ^2^ test). While it needs to be addressed in the future how commonly and specifically shared and clustered clonotypes are present in peripheral blood of patients with T1D, these results suggest the presence of public and potentially disease-specific TCR clonotypes in pancreatic tissues that could be used as biomarkers to differentiate individuals with T1D.

## Discussion

TCR clonotype tracing in the peripheral blood may serve as a potential biomarker for autoimmune diseases such as T1D (29, 30). We demonstrated the presence of “public” TCR clonotypes commonly expressed by T cells in the pancreas of multiple individuals in both humans and mice. These public TCR clonotypes were quantitatively detectable in blood samples of NOD mice using V gene–targeted sequencing. This method effectively distinguished T1D-susceptible NOD mice from those protected from T1D development, especially when including TCRs similar to the initially identified public TCR in enumeration. In the TCR sequencing analysis of human pancreatic samples at various stages of T1D, we identified public TCR clonotypes expressed by islet-antigen-specific T cells and therefore likely to be disease specific, but also those commonly present regardless of disease status. This underscores the importance of discriminating TCR clonotypes mirroring disease-specific T cell activity in the pancreas.

Previous studies have shown that TCR clonotypes expressed by T cells in the pancreas are detected in PLNs and the blood of the same individuals (31, 32). Our current study provides further evidence that TCR clonotypes frequent in the islets have a higher chance of being traced from the islets to PLNs to blood, indicating that the proportion of TCR repertoires is conserved from the islets to the blood. Thus, quantitative assessment of islet-associated TCR clonotypes in the blood may allow us to grasp T cell repertoires in the islets. However, quantifying autoimmune TCR clonotypes in blood samples is challenging due to the infrequency of antigen-specific T cells, typically accounting for less than 0.1% (6). Particularly, self-reactive T cells are extremely rare compared with those specific for pathogens such as common viruses and vaccine antigens. Nevertheless, investigators have been attempting to develop TCR sequencing assays to quantitatively evaluate the frequencies of TCR clonotypes specific to disease statuses, and several assays have successfully differentiated individuals infected with certain viruses from those without, which correlated with clinical disease severity (33–36). Quantifications in these assays evaluate differences in clonotype frequencies ranging between 0.01% and 0.1%. To overcome even more challenging infrequencies expected in autoreactive TCR clonotypes, we employed V gene–targeted sequencing, assessing only TCRs containing a specific V gene. This approach results in frequencies of evaluated TCR clonotypes tens of times higher than those in TCR sequencing assays examining TCRs with any V genes. In this proof-of-principle study focusing on only TCRs with a specific V gene, frequencies of TCR clonotypes expressed by islet-antigen-specific CD8^+^ T cells were assessed in the range between 0.1% and 10%, allowing for clear discrimination of animals developing diabetes. Previous studies using fluorescent protein–conjugated peptide-MHC multimers to quantify IGRP peptide–specific T cells in the blood have reported pronounced temporal and interanimal variability in their frequencies (37). Consistent with these findings, our study demonstrates that the TCR clonotypes expressed by these T cells also fluctuate both within and between individual animals. These results underscore the potential of V gene–targeted TCR clonotype analysis as a surrogate non–cell-based biomarker for monitoring antigen-specific T cell dynamics.

For V gene–targeted TCR sequencing, it is essential to identify V genes to examine TCR clonotypes specific to an antigen of interest. TCRs recognizing the same epitope often share the same V gene, such as TRBV19 in TCR clonotypes specific to an influenza virus matrix protein epitope (38, 39). In T cells specific for T1D-associated antigens, a significant portion of IGRP:206–214-specific CD8^+^ T cells in NOD mice express TCR clonotypes with the TRAV16/16D V-α genes, as highlighted in this study (17, 23–25). Similarly, insulin B chain–specific CD4^+^ T cells in NOD islets frequently use TRAV5D-4 (19, 40–42), and human IGRP/HLA-A2–specific TCRs preferentially use TRAV29 (20). Thus, TCRs often recognize the same epitopes by TCRs with the same V genes, which contain the CDR1 and CDR2 regions to interact with a peptide-MHC complex, allowing V gene–targeted sequencing. It is notable that such common V gene motifs for islet antigens are often identified in V-α genes. A recent study analyzing TCR clonotypes expressed by hybrid insulin peptides, fusion peptides consisting of a proinsulin peptide at the N-terminus and an islet protein–derived peptide at the C-terminus (43), suggested that the N- and C-terminal peptides are primarily recognized by each α and β TCR chain, respectively (44). As hybrid insulin peptides often share the same N-terminal proinsulin peptide, which may be predominantly recognized by an α chain, specific V-α genes might be preferentially used by TCR clonotypes recognizing islet tissues. We found that public TCR clonotypes in the islets were dominantly found in α chains in both NOD mice and T1D organ donors. Linsley and Cerosaletti also reported that common motifs recognizing islet antigens are prominently identified in TCR α chains as well (45). While most TCR sequencing studies currently focus on β chain sequences, these observations underscore the importance of including α chain sequences in TCR studies for T1D. As observed in TCR clonotypes specific for hybrid insulin peptides that preferentially use a combination of TRAV26-1 and TRBV5 (44), integrating both α and β V gene–targeted sequencing data may enhance the identification of disease-specific TCR clonotypes. Such a combined approach could provide a more comprehensive view of the T cell repertoire in T1D and potentially improve the sensitivity and specificity of TCR-based biomarkers.

Since self-reactive T cells are rare in the blood, it is important to have as many known traceable TCR clonotypes as possible to maximize the detection of self-reactive ones. As only a quarter of islet-antigen-specific TCR clonotypes are public (30), identifying islet-specific TCR clonotypes that can be commonly traced in blood samples of a certain portion of individuals will be crucial for the successful development of TCR biomarkers (4). To this end, a TCR clustering strategy may be beneficial to increase traceable TCR clonotypes as islet-antigen-specific ones in the blood. Indeed, when including TCR clonotypes similar to the NY8.3 TCR (i.e., extended NY8.3) to enumerate IGRP-specific T cells, additional animals developing T1D were able to be identified. Computational TCR clustering methods have been developed to predict antigen-specific TCR clonotypes, and their prediction specificities are continuously improved (28, 46–49). A recent study demonstrated that the development of high-grade serious ovarian cancer can be predicted 2 years before clinical onset by quantitatively evaluating the presence of TCR clonotypes included in only 4 clusters (50). Additionally, parsing full TCR α and β chain sequences into partial motifs may enhance the identification of shared TCR sequence features, as recently shown in a large-scale analysis of peripheral blood samples from individuals with and without T1D (51). Thus, while there are relatively limited numbers of common TCR clonotypes detected from human pancreatic tissues, integrating these computational clustering tools may be beneficial to improve sensitivity of TCR sequencing assays for the prediction of autoimmune diseases.

A major difference between the NOD model and humans is the greater MHC diversity in humans. NOD mice express only 1 class II molecule (I-A^g7^) and 2 class I molecules (K^d^ and D^b^), whereas humans may express up to 12 HLA class II molecules (including DQ and DP α-β *trans*-combinations) (52) and as many as 8 class I molecules when including HLA-E (53). This diversity complicates identification of disease-associated TCR clonotypes in humans. While the number of clonotypes obtained from human pancreatic tissues was too limited to assess the influence of HLA matching on clonotype sharing, future analyses restricted to individuals with the same HLA alleles or haplotypes may help reduce noise introduced by responses to unrelated antigens. Given the strong genetic association with HLA-DR4 and -DR3 haplotypes (52), clonotypes consistently observed in individuals with these T1D-risk HLAs but absent in those without may more reliably indicate disease association. Alternatively, computational approaches predicting HLA restriction from TCR motifs, such as grouping clonotypes predicted to bind HLA-DQ8, may also be useful (54). To advance the field, expanding TCR datasets from both T1D and non-T1D individuals will be essential to build a robust database of disease-associated clonotypes measurable in blood.

In this study, we used the NY8.3 clonotype, expressed by CD8^+^ T cells specific for IGRP:206–214, as a model to evaluate the potential of TCR clonotypes for quantitative biomarker development. IGRP:206–214-specific T cells expand during later stages of T1D in NOD mice, coinciding with extensive immune infiltration in the islets (55), which is consistent with our observation that NY8.3 clonotypes were frequently detected in islets. These findings suggest that quantifying islet-specific TCR clonotypes may be more feasible in later disease stages. On the other hand, T cells involved in the initiation of autoimmunity may be relatively infrequent. Given the strong genetic risk association within the HLA class II locus, CD4^+^ T cells are likely to play a key role early in islet autoimmunity. It remains unknown whether these initiating cells expand sufficiently and traffic into the circulation. Future studies will be necessary to determine whether TCR-based biomarkers can detect T cells contributing to the earlier phases of T1D development.

In future studies, our goal is to apply V gene–targeted TCR sequencing to track T1D-associated clonotypes across distinct disease stages in humans, with the dual purpose of advancing quantitative biomarker development and elucidating antigen-specific T cell dynamics during disease progression. In our previous work, we showed that TCR clonotypes specific for islet antigens identified from T1D patients were preferentially detected in a cohort of patients with new-onset T1D (30). Building on this, V gene–targeted TCR sequencing will allow us to quantitatively measure the frequencies of islet-antigen-specific clonotypes and to determine, at the individual level, which antigen-specific clonotypes appear earlier or later during disease progression. Importantly, a key advantage of this approach is that it does not require prior knowledge of epitopes, unlike conventional antigen-specific T cell assays such as peptide-MHC multimer analysis. This feature opens the possibility of detecting responses to epitopes that have not been characterized in existing assays but may be critical for initiating or driving β cell autoimmunity in T1D. Together, our results establish a foundation for future studies using V gene–targeted TCR sequencing to track disease-associated clonotypes in humans.

We have demonstrated that the presence of autoreactive T cells can be quantitatively assessed by V gene–targeted TCR sequencing, in which TCR clonotypes specific for self-antigens may be enriched by evaluating only TCRs containing specific V genes. This method can discriminate prediabetic and diabetic animals from those protected from T1D development. To apply the method to human T1D, identifying sufficient human islet-antigen-specific TCR clonotypes that can be commonly and specifically traced in the blood of patients will be crucial.

## Methods

### Sex as a biological variable.

Our study exclusively examined female mice because diabetes incidence in females is significantly higher than that in male mice.

### Mice and diabetes monitoring.

Female NOD/ShiLtJ mice (stock 001976) were purchased from The Jackson Laboratory. Insulin-KO NOD mice, lacking functional *Ins1* and *Ins2* genes but transgenic for a mutated preproinsulin gene at insulin B chain position 16 (The Jackson Laboratory, stock 005525) (26), were housed at the University of Colorado Anschutz Medical Campus. For longitudinal TRAV16 sequencing studies, female wild-type NOD and insulin-KO NOD mice were sampled every 5 weeks from 5 weeks of age until diabetes onset or until 20 weeks of age. Blood glucose levels were monitored using a ReliOn Ultima Blood Glucose meter (Abbott Diabetes Care, Inc.), with diabetes onset defined by 2 consecutive readings above 250 mg/dL across 2 days.

### Tissue collection and RNA isolation.

Approximately 200 μL of blood was collected from each mouse, followed by RBC lysis (Sigma-Aldrich). Islets were isolated by perfusing the pancreas with collagenase (Sigma-Aldrich), enriched on a Lympholyte (Cedarlane) density gradient, and manually picked (56). PLNs and spleen were surgically removed postmortem and processed to obtain single-cell suspensions. RNA was extracted using the Qiagen RNeasy Mini Kit and used immediately for cDNA synthesis.

### Whole TCR sequencing.

Whole TCR sequencing was adapted from protocols previously described (57). Briefly, cDNA was synthesized using the SMARTer RACE cDNA amplification kit with 5′RACE CDS primer A and SMARTer II A oligonucleotide (Takara). TCR α and β chain genes were amplified through 2 rounds of nested PCR, with the first round using primers for the TCR constant region and universal primers (Supplemental Table 1). The products underwent a second PCR with nested primers containing sequencing adapters and barcodes (Supplemental Table 1). The amplified products were sequenced on either a 454 GS Junior (Roche), an Illumina MiSeq, or an Illumina NovaSeqX system. Sequence data for cohort 1 samples generated using the 454 GS Junior platform (Supplemental Table 3) were demultiplexed according to DNA barcode identifiers and processed using the IMGT HighV-QUEST algorithm (https://www.imgt.org/) to identify TCR clonotypes. Sequence data for cohorts 2 and 3 samples generated using the Illumina NovaSeq X platform (Supplemental Table 3) were analyzed using MiXCR (https://mixcr.com) (58). MiSeq-derived sequences from islets, pancreatic lymph nodes, and blood samples (Supplemental Table 5) were demultiplexed by sample-specific barcode identifiers, and reads sharing identical unique molecular identifiers (UMIs) were collapsed to represent unique sequences prior to downstream analysis. These sequences were then processed using IMGT HighV-QUEST. For IMGT-based analyses, TCR clonotypes were defined by unique combinations of CDR3 amino acid sequence and assigned V and J gene segments. Only productive rearrangements with high-confidence V-gene assignments (V-gene score >1200, indicative of ≥95% homology to reference sequences) were included. For each sample, clonotype counts reported by IMGT were summed to obtain total TCR counts, and the relative frequency of each clonotype was calculated by normalizing individual clonotype counts to the total count per sample. Clonotype count and frequency matrices were generated separately for TCR α and β chains, and clonotype sharing across samples and tissues was assessed by comparing clonotypes with identical CDR3 amino acid and V/J gene assignments.

### TRAV16-targeted sequencing.

cDNA was synthesized using random hexamers, followed by 2 rounds of nested PCR specifically amplifying TCR α chains containing the TRAV16 V gene segment using primers specific for TRAV16 and the constant region of TCR α chains (Supplemental Table 2) for sequencing. Sequencing data were demultiplexed and analyzed for clonotype frequencies using MiTCR (58) or MiXCR (59).

### Reactivity testing of TCR clonotypes.

TCR α (public NY8.3) and β chain gene fragments frequently detected in NOD islets were cloned into murine stem cell virus–based (MSCV-based) retroviral vectors (60). Retroviruses produced from the public NY8.3 α chain and grouped or single β chain genes were used to transduce 5KC hybridoma T cells lacking expression of endogenous TCR α and β chains, which were provided by John Kappler (National Jewish Health, Denver, Colorado, USA) (61), by spinfection. Transduced cells were isolated by flow sorting of cells stained with an anti-CD3 antibody (clone 145-2C11, BD Biosciences). 5KC TCR transductants (100,000 cells per well) were cultured overnight with or without peptides at 100 μg/mL or anti-CD3 antibody (clone 145-2C11) at 5 μg/mL in the presence of NOD spleen cells (100,000 cells per well). IL-2 secreted into the culture supernatant was measured by ELISA using a capture antibody against IL-2 (clone JES6-1A12, BD Biosciences) and a biotinylated detection antibody (clone JES6-5H4, BD Biosciences), followed by europium-labeled streptavidin and DELFIA enhancement solution (PerkinElmer), with time-resolved fluorescence detection. Peptides tested for reactivity by TCR transductants include an IGRP 206–214 peptide (VYLKTNVFL) (37), an insulin B chain 9–23 peptide (SHLVEALYLVCGERG) (62), a mimotope for diabetogenic T cell clone BDC2.5 (pS3, SRLGLWVRME) (63), and a negative control peptide presented by H2-K^d^ (TUM, KYQAVTTTL) (37). All peptides were synthesized by Genemed Synthesis Inc.

### Identification of TCR clonotypes in human islets.

Islet and pancreas slice samples were provided from the Network for Pancreatic Organ Donors with Diabetes program (nPOD; RRID:SCR_014641) (64), a protocol at Vanderbilt University Medical Center/University of Pittsburgh (65), the Integrated Islet Distribution program (IIDP; RRID:SCR_014387), and the Alberta Diabetes Institute IsletCore (ADI). T cells were isolated from islet or pancreas slice samples, and TCR α and β chain sequences were identified from individual T cells, as described previously (66). TCR clonotypes of individual DNA fragments were identified using the IMGT HighV-QUEST algorithm. Clonotype overlap between individual donors was analyzed using an R-based algorithm (https://github.com/CUAnschutzBDC/maki_tcr_sequence_analysis), and TCRdist (28) was used for clustering analysis.

### Statistics.

Data analyses were conducted using Prism 9 software (GraphPad Software) and R software (R Core Team). The specific statistical tests applied are indicated in the respective text, table, or figure legend. *P* values less than 0.05 were considered statistically significant.

### Study approval.

All mouse studies were approved by the Institutional Animal Care and Use Committee at the University of Colorado Anschutz Medical Campus. Human TCR sequencing studies were performed as Not Human Subject Research determined by the Colorado Multiple Institutional Review Board at the University of Colorado Anschutz Medical Campus. Islet isolation was approved by the Human Research Ethics Board at the University of Alberta (Pro00013094). All donors’ families gave informed consent for the use of pancreatic tissue in research.

### Data availability.

All data sets generated or analyzed during this study have been included in this manuscript, and values for all data points in graphs are reported in the Supporting Data Values file.

## Author contributions

MN and AWM provided essential concepts and designed the studies. LGL and AMA conducted experiments, and LGL, KLW, KLJ, and MN analyzed data. KRM performed statistical analysis. LGL and MN wrote the manuscript, and all authors reviewed and edited the manuscript.

## Funding support

This work is the result of NIH funding, in whole or in part, and is subject to the NIH Public Access Policy. Through acceptance of this federal funding, the NIH has been given a right to make the work publicly available in PubMed Central.

- National Institutes of Diabetes and Digestive and Kidney Diseases grants R01DK099317, R01DK032083, R01DK133457, R01DK108868 (all to MN and AWM), P30DK116073 (to the University of Colorado Diabetes Research Center), and 2UC4DK098085 and U24DK098085 (both to the IIDP).
- National Cancer Institute grant P30CA046934 (to the University of Colorado Cancer Center).
- Breakthrough T1D grants 2-SRA-2018-480-S-B (to MN and AWM), 1-SRA-2020-911-A-N (to MN), 3-SRA-2022-1248-S-B (to MN and AWM), and 5-SRA-2018-557-Q-R (to the nPOD).
- Leona M. and Harry B. Helmsley Charitable Trust grant 2301-06562 (to MN and AWM).

## Supplementary Material

Supplemental data

Supporting data values

## Figures and Tables

**Table 1:**
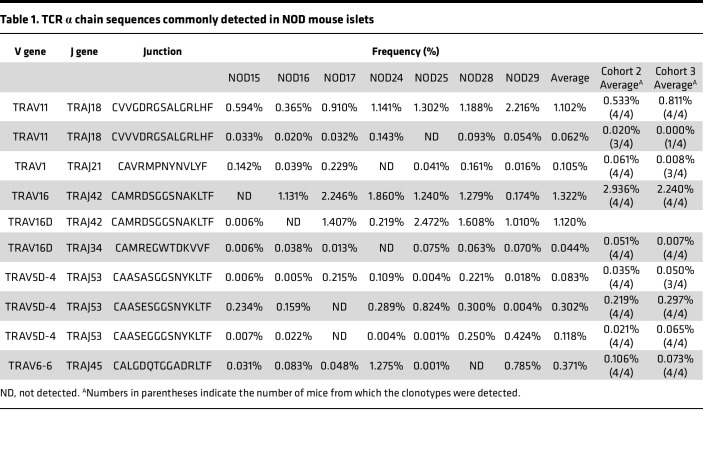
TCR α chain sequences commonly detected in NOD mouse islets

**Table 2:**
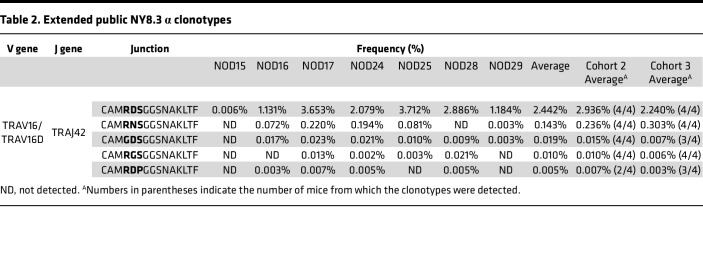
Extended public NY8.3 α clonotypes

## Version 1 12/18/2025

In-Press Preview

## Version 2 02/09/2026

Electronic publication
