# Supplementary material for: Quantitative V gene–targeted T cell receptor sequencing as a biomarker in type 1 diabetes
Source: JCI Insight. 2025 Dec 18;11(3):e186004. doi: 10.1172/jci.insight.186004 (PMC12893103; doi:10.1172/jci.insight.186004)
Supplement: Supplemental data [file jciinsight-11-186004-s241.pdf]

Supplemental Figure 1

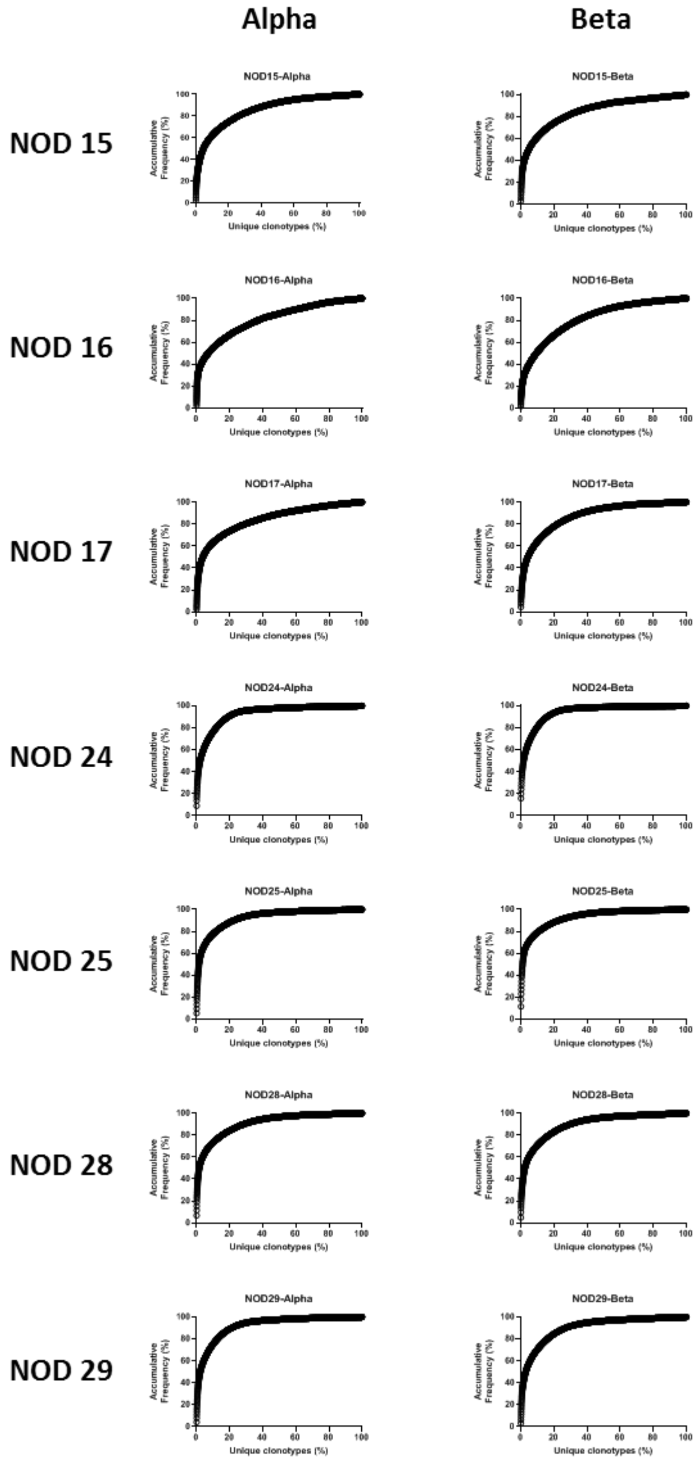

Supplemental Figure 1: Accumulative frequency of unique TCR clonotypes. Each graph depicts TCR alpha or beta repertoires in the islets of individual NOD mice studied. Individual unique TCR clonotypes are aligned in X-axis (highest to lowest clonotypes), and the accumulative frequencies of all clonotypes that are more frequent than any given clonotypes are plotted in Y-axis. Sharp slopes in the first few percent of unique clonotypes demonstrate the deflect prevalence of those clonotypes.

Supplemental Figure 2

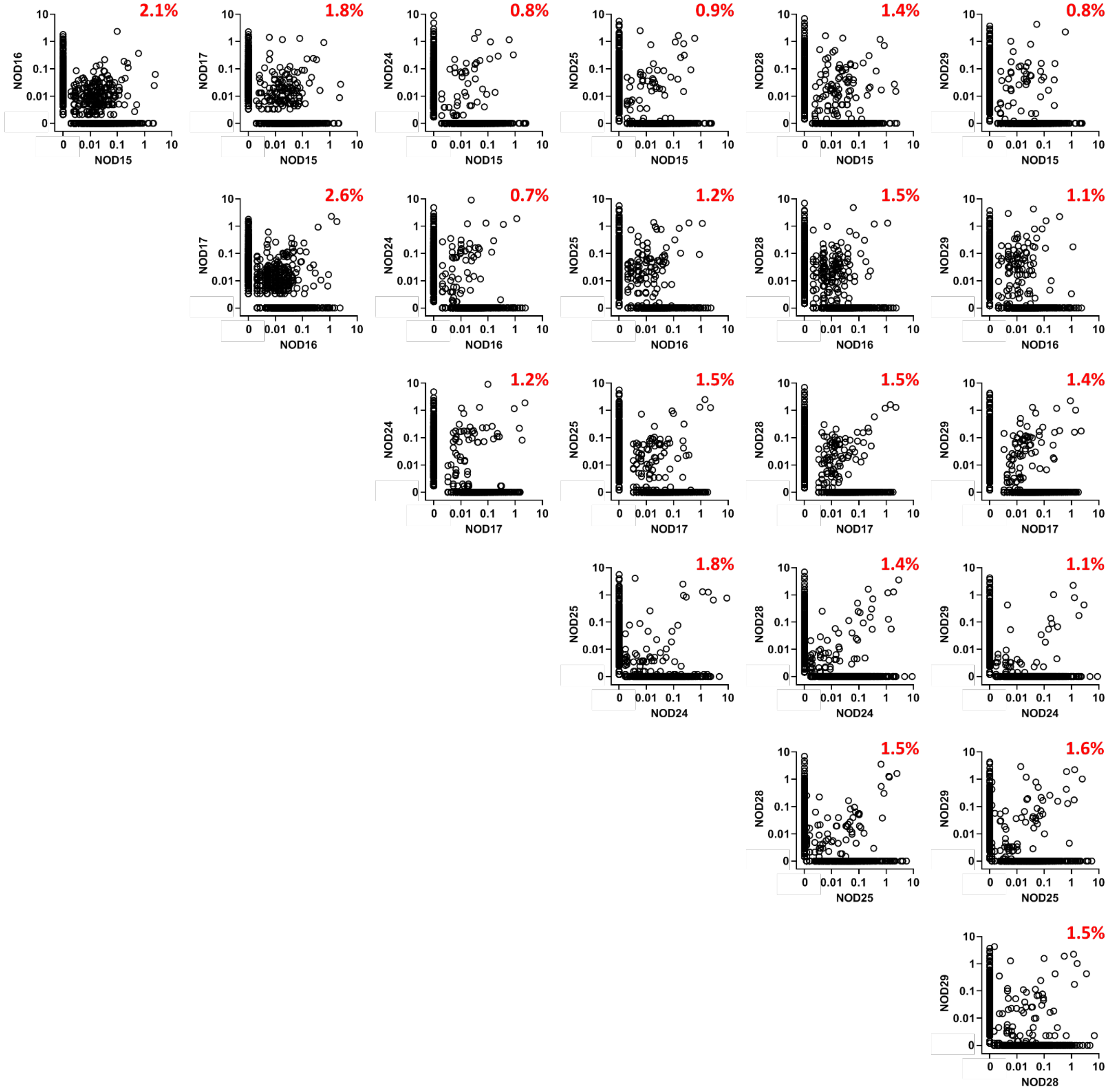

Supplemental Figure 2: Comparison of TCR alpha clonotype frequencies in the islets. Each graph depicts TCR alpha repertoires in the islets of any two given NOD mice studied of the 21 possible pairings. Symbols represent frequency of each unique clonotype of one mouse in X-axis and that of the other mouse in Y-axis. Percentages of clonotypes that were detected in the islets of both mice are shown in red at the right-top corner of each graph.

Supplemental Figure 3

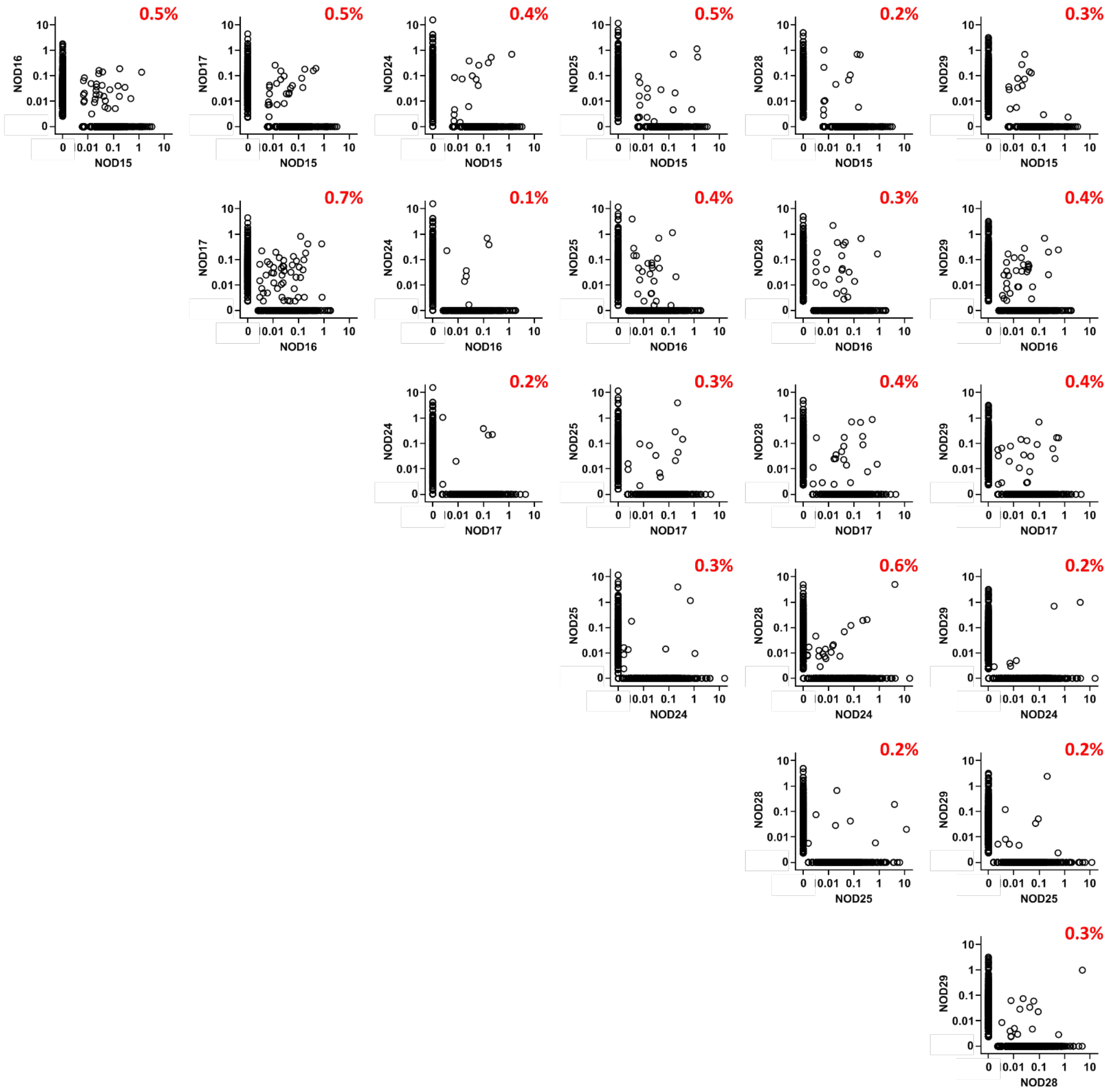

Supplemental Figure 3: Comparison of TCR beta clonotype frequencies in the islets. Each graph depicts TCR beta repertoires in the islets of any two given NOD mice studied of the 21 possible pairings. Symbols represent frequency of each unique clonotype of one mouse in X-axis and that of the other mouse in Y-axis. Percentages of clonotypes that were detected in the islets of both mice are shown in red at the right-top corner of each graph.

Supplemental Figure 4

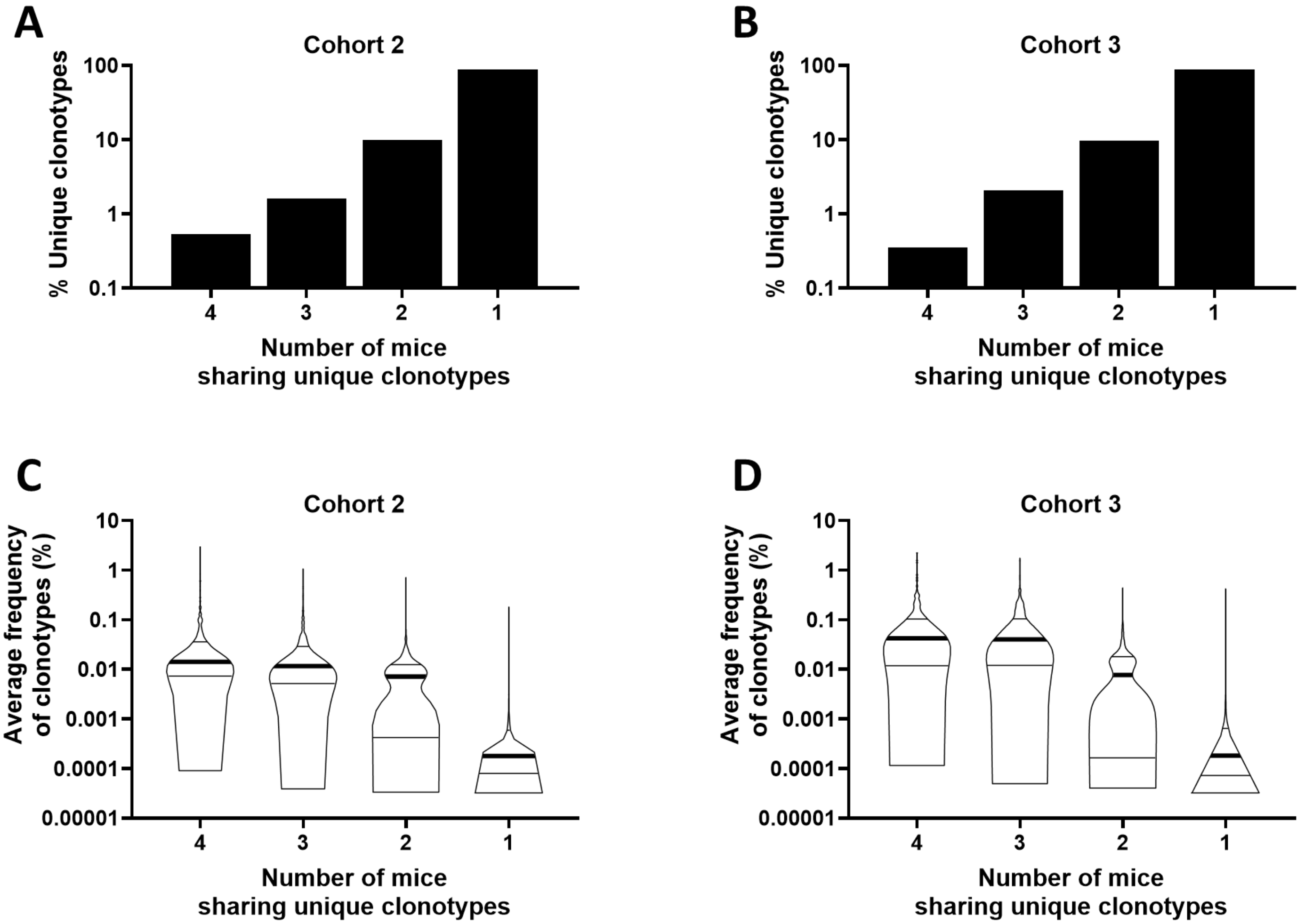

Supplemental Figure 4: Frequencies of TCR clonotypes shared between animals in cohorts 2 and 3. (A) and (B) Percentages of TCR alpha clonotypes detected from different numbers of mice are plotted. Approximately 90% of clonotypes were detected from only a single mouse. (C) and (D) Frequencies of TCR alpha clonotypes detected from different numbers of mice are shown in violin plots. Frequencies of private clonotypes (i.e. detected from only a single mouse) are lower than public clonotypes (i.e. detected from multiple mice).

Supplemental Figure 5

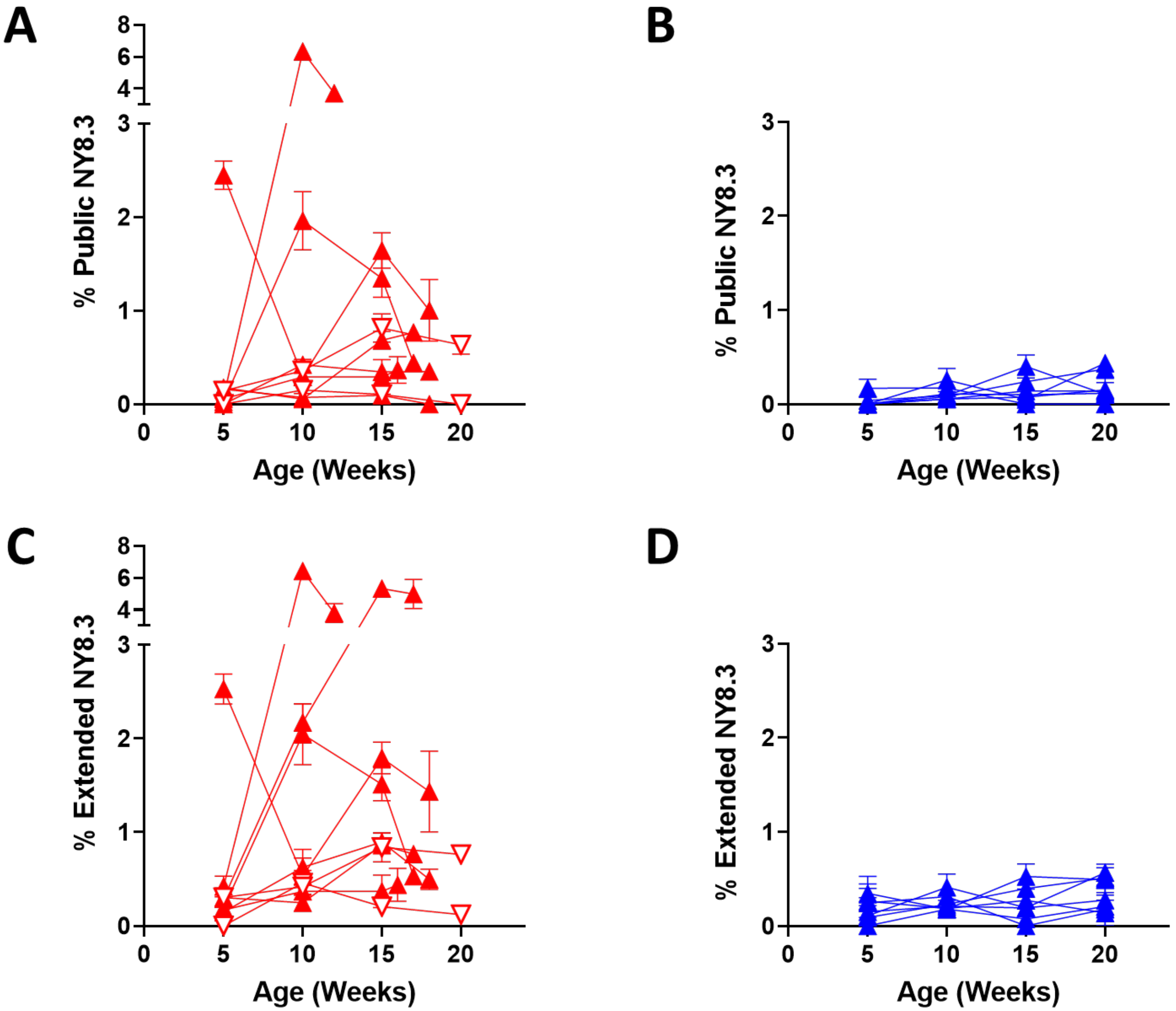

Supplemental Figure 5: Longitudinal analysis of frequencies of public and extended NY8.3 clonotypes. Frequencies of public NY8.3 (A and B) and extended NY8.3 clonotypes (C and D) in blood samples of nine NOD (A and C) and seven insulin-knockout NOD mice were determined using TRAV16-targeted sequencing. Symbols connected by lines represent data from each mouse. White inverse triangle symbols in panels A and C represent data from NOD mice that did not develop diabetes during the study period.

Supplemental Figure 6

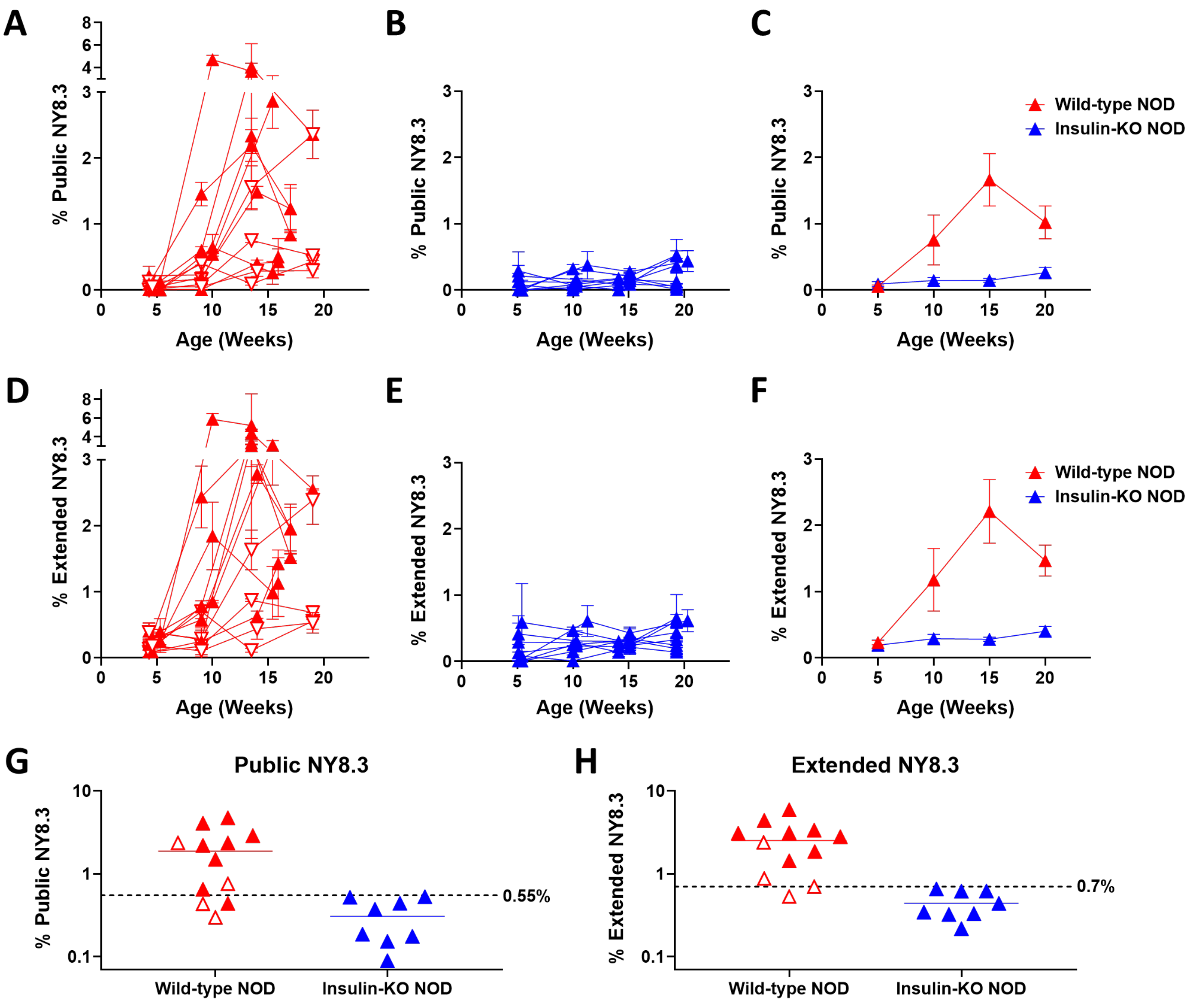

Supplemental Figure 6: Longitudinal analysis of frequencies of public and extended NY8.3 clonotypes of the second cohorts. Frequencies of public NY8.3 (A and B) and extended NY8.3 clonotypes (D and E) in blood samples of a cohort of 12 NOD (A and D) and 8 insulin-knockout NOD mice were determined using TRAV16-targeted sequencing. Mean values ± standard errors of the public NY8.3 and extended NY8.3 clonotypes of these cohorts are shown in panels C and F, respectively. The highest values of individual mice during the study period are plotted for public NY8.3 (G) and extended NY8.3 (H). The dashed line is the cut-off value determined by the 99 percentile of highest values in insulin-KO mice. White inverse triangle symbols in panels A, D, G, and H represent data from NOD mice that did not develop diabetes during the study period.

Supplemental Figure 7

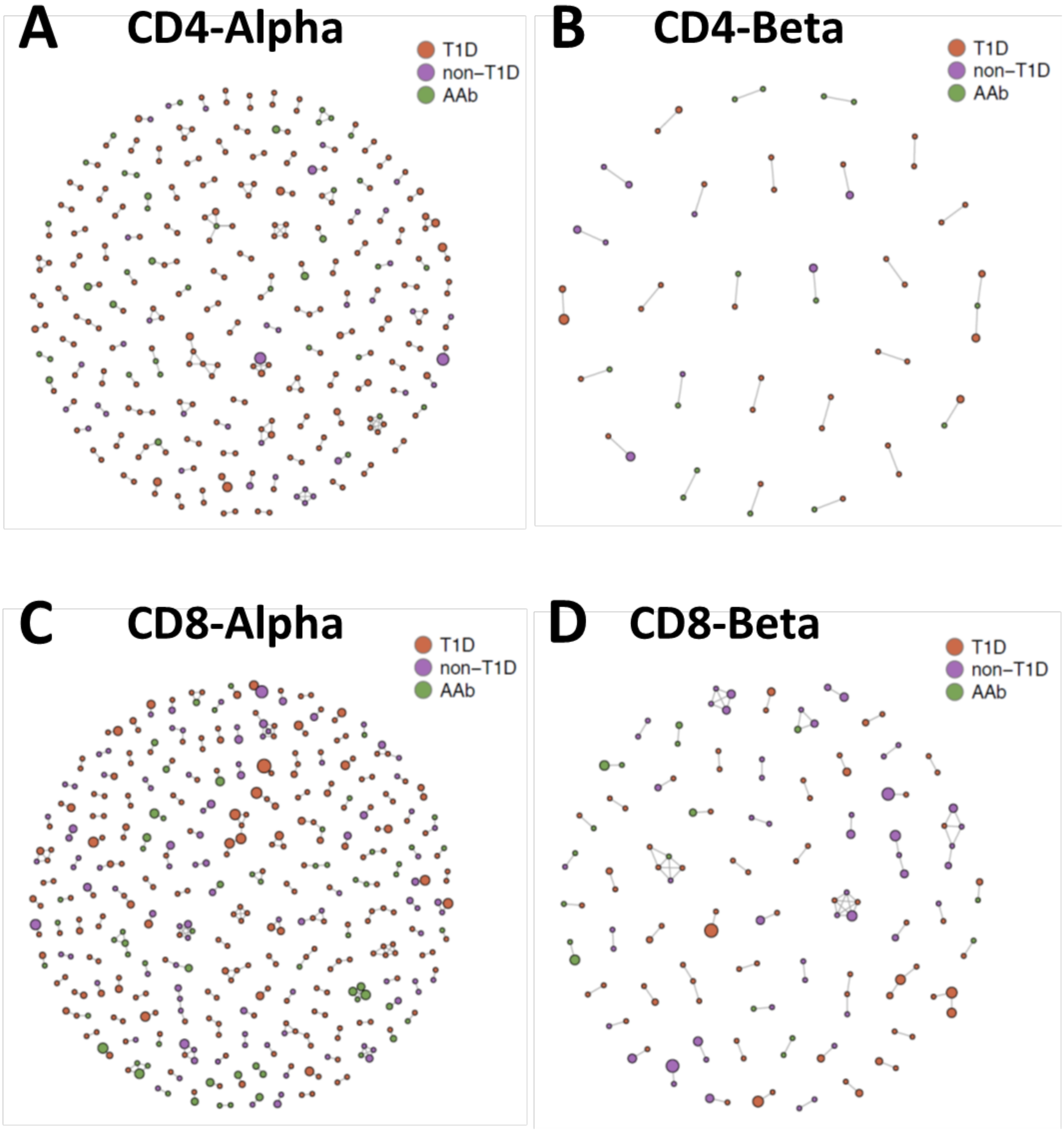

Supplemental Figure 7: Clusters of TCR clonotypes. TCR clonotypes detected from islets and pancreas tissues were analyzed for similarity using TCRdist. Each bubble represents a unique clonotype from each donor, which is connected to clonotypes having similar motifs. Size of bubbles represents the number of cells expressing a give clonotype.

Supplemental Table 1

Supplemental Table 1: Primers to generate whole TCR amplicons

Alpha chain PCR products sequenced on 454 GSJR

1st step PCR	Primer Name	Primer Sequence
**Primer 1**	MAC	GGGTGCTGTCCTGAGACCGAGGATC
**Primer 2**	Universal Primer A Mix	Provided from SMARTer RACE cDNA amplification kit (Takara)
**2nd step PCR**	**Primer Name**	**Primer Sequence**
**Primer 1**	LibL_MIDsX_MAC	CCATCTCATCCCTGCGTGTCTCCGACTCAG (454 adapter)-XXXXX (barcode)-GTACACAGCAGGTTCTGGGTTCTGG
**Primer 2**	TitB-RACE	CCTATCCCCTGTGTGCCTTGGCAGTCTCAG (454 adapter)-AAGCAGTGGTATCAACGCAGAGT

Alpha chain PCR products sequenced on Illumina MiSEQ

1st step PCR	Primer Name	Primer Sequence
**Primer 1**	MAC	GGGTGCTGTCCTGAGACCGAGGATC
**Primer 2**	Illumina-UMI	CAGACGTGTGCTCTTCCGATCT-NNNNNNNN (random 6-8mers)-AAGCAGTGGTATCAACGCAGAGT
**Primer 3**	Illumina-short	CAGACGTGTGCTCTTCCGATCT
**2nd step PCR**	**Primer Name**	**Primer Sequence**
**Primer 1**	Illumina-MAC-MIDsY	AATGATACGGCGACCACCGAGATCT (Illumina adapter)-ACACTCTTTCCCTACACGACGCTCTTCCGATCT (Illumina sequencing primer sequence)-YYYYYYY (barcode)-GTACACAGCAGGTTCTGGGTTCTGG
**Primer 2**	Illumina-IndexZ	CAAGCAGAAGACGGCATACGAGAT (Illumina adapter)-ZZZZZZ (barcode)- GTGACTGGAGTTCAGACGTGTGCTCTTCCGATCT (Illumina sequencing primer sequence)

Beta chain PCR products sequenced on 454 GSJR

1st step PCR	Primer Name	Primer Sequence
**Primer 1**	MBC3	AGCCCATGGAACTGCACTTGGCAGCG
**Primer 2**	Universal Primer A Mix	Provided from SMARTer RACE cDNA amplification kit (Takara)
**2nd step PCR**	**Primer Name**	**Primer Sequence**
**Primer 1**	LibL_MIDsX_MBC3	CCATCTCATCCCTGCGTGTCTCCGACTCAG (454 adapter)-XXXXX (barcode)- CCTGGCCAAGCACACGAGGG
**Primer 2**	TitB-RACE	CCTATCCCCTGTGTGCCTTGGCAGTCTCAG (454 adapter)-AAGCAGTGGTATCAACGCAGAGT

Beta chain PCR products sequenced on Illumina MiSEQ

1st step PCR	Primer Name	Primer Sequence
**Primer 1**	MBC3	AGCCCATGGAACTGCACTTGGCAGCG
**Primer 2**	Illumina-UMI	CAGACGTGTGCTCTTCCGATCT-NNNNNNNN (random 6-8mers)-AAGCAGTGGTATCAACGCAGAGT
**Primer 3**	Illumina-short	CAGACGTGTGCTCTTCCGATCT
**2nd step PCR**	**Primer Name**	**Primer Sequence**
**Primer 1**	Illumina-MBC-MIDsY	AATGATACGGCGACCACCGAGATCT (Illumina adapter)-ACACTCTTTCCCTACACGACGCTCTTCCGATCT (Illumina sequencing primer sequence)-YYYYYYY (barcode)-ACAAGGAGACCTTGGGTGGAGTCAC
**Primer 2**	Illumina-IndexZ	CAAGCAGAAGACGGCATACGAGAT (Illumina adapter)-ZZZZZZ (barcode)- GTGACTGGAGTTCAGACGTGTGCTCTTCCGATCT (Illumina sequencing primer sequence)

Supplemental Table 2

Supplemental Table 2: Primers to generate TRAV16-TCR amplicons

1st step PCR	Primer Name	Primer Sequence
**Primer 1**	TRAV16-F2	ACGGTGACAATGGACTGTGTGTATG
**Primer 2**	MAC short	GTGCTGTCCTGAGACCGAGGATC
**2nd step PCR**	**Primer Name**	**Primer Sequence**
**Primer 1**	TRAV16-Illumina-1	CAAGCAGAAGACGGCATACGAGAT (Illumina adapter)- CGTGAT (barcode)- GTGACTGGAGTTCAGACGTGTGCTCTTCCGATCT (Illumina sequencing primer sequence)- TGGTACAAGCAAACAGCAAGTGG
	TRAV16-Illumina-3	CAAGCAGAAGACGGCATACGAGAT (Illumina adapter)- GCCTAA (barcode)- GTGACTGGAGTTCAGACGTGTGCTCTTCCGATCT (Illumina sequencing primer sequence)- ACTGGTACAAGCAAACAGCAAGTGG
	TRAV16-Illumina-4	CAAGCAGAAGACGGCATACGAGAT (Illumina adapter)- TGGTCA (barcode)- GTGACTGGAGTTCAGACGTGTGCTCTTCCGATCT (Illumina sequencing primer sequence)- AACTGGTACAAGCAAACAGCAAGTGG
	TRAV16-Illumina-6	CAAGCAGAAGACGGCATACGAGAT (Illumina adapter)- ATTGGC (barcode)- GTGACTGGAGTTCAGACGTGTGCTCTTCCGATCT (Illumina sequencing primer sequence)- AACTGGTACAAGCAAACAGCAAGTGG
	TRAV16-Illumina-7	CAAGCAGAAGACGGCATACGAGAT (Illumina adapter)- GATCTG (barcode)- GTGACTGGAGTTCAGACGTGTGCTCTTCCGATCT (Illumina sequencing primer sequence)- CTGGTACAAGCAAACAGCAAGTGG
	TRAV16-Illumina-8	CAAGCAGAAGACGGCATACGAGAT (Illumina adapter)- TCAAGT (barcode)- GTGACTGGAGTTCAGACGTGTGCTCTTCCGATCT (Illumina sequencing primer sequence)- CTGGTACAAGCAAACAGCAAGTGG
	TRAV16-Illumina-9	CAAGCAGAAGACGGCATACGAGAT (Illumina adapter)- CTGATC (barcode)- GTGACTGGAGTTCAGACGTGTGCTCTTCCGATCT (Illumina sequencing primer sequence)- CTGGTACAAGCAAACAGCAAGTGG
	TRAV16-Illumina-10	CAAGCAGAAGACGGCATACGAGAT (Illumina adapter)- AAGCTA (barcode)- GTGACTGGAGTTCAGACGTGTGCTCTTCCGATCT (Illumina sequencing primer sequence)- CTGGTACAAGCAAACAGCAAGTGG
**Primer 2**	Illumina-MAC-MIDsY	AATGATACGGCGACCACCGAGATCT (Illumina adapter)- ACACTCTTTCCCTACACGACGCTCTTCCGATCT (Illumina sequencing primer sequence)-YYYYYYY (barcode)- GTACACAGCAGGTTCTGGGTTCTGG

Supplemental Table 3

Supplemental Table 3: TCR sequencing of NOD mouse islets

Cohort*	Mouse ID	Age at sampling (weeks)	Litter information	Nb of islets processed	Total alpha in-frame reads	Unique alpha in-frame clonotypes	Total beta in-frame reads	Unique beta in-frame clonotypes
1	NOD15	8.5	A	115	38,137	4,232	15,142	2,424
1	NOD16	7.2	B	45	57,583	7,297	29,150	4,528
1	NOD17	7.2	B	100	19,539	3,763	30,039	2,528
1	NOD24	8.1	C	130	41,988	1,849	52,980	1,295
1	NOD25	8.1	C	120	45,449	2,243	38,851	1,468
1	NOD28	9.3	D	125	50,747	2,759	36,937	2,514
1	NOD29	9.3	D	125	51,691	2,348	35,254	2,359
2	NOD2025-1	9.7	2025-A	185	4,445,366	13,113	Not done	
2	NOD2025-2	9.3	2025-B	185	4,717,345	15,607		
2	NOD2025-3	10.4	2025-C	135	3,045,407	13,375		
2	NOD2025-4	10.4	2025-C	190	4,338,267	15,582		
3	NOD2025-5	16.1	2025-B	70	3,517,206	11,005		
3	NOD2025-6	10.1	2025-D	175	3,596,129	9,856		
3	NOD2025-7	9.0	2025-E	110	4,108,620	7,082		
3	NOD2025-8	9.0	2025-E	200	2,403,250	4,833		

* Cohort 1: animals analyzed for the results shown in Figure 1 and Tables 1 and 2.
Cohort 2 and 3: animals analyzed for the results shown in Tables 1 and 2.

Supplemental Table 4

Supplemental Table 4: Beta clonotypes tested for the paring with public NY8.3 alpha

Beta ID	TRBV	TRBJ	AA JUNCTION	% in NOD24	Rank in NOD24	% in NOD25	Rank in NOD25	Number of mice sharing
NOD24-B1	TRBV3	TRBJ2-1	CASSPGTGGEDAEQFF	15.72%	1	ND		1
NOD24-B2	TRBV13-1	TRBJ2-1	CASSDRGVAEQFF	4.21%	2	ND		1
NOD24-B3	TRBV15	TRBJ2-7	CASSRDSSYEQYF	4.11%	3	ND		3
NOD24-B4	TRBV15	TRBJ2-7	CASSFSGDEQYF	3.19%	4	ND		1
NOD24-B5	TRBV15	TRBJ2-7	CASSLAQYEQYF	2.85%	5	ND		1
NOD24-B6	TRBV13-3	TRBJ1-4	CASSDAGVNERLFF	2.77%	3	ND		1
NOD24-B7	TRBV4	TRBJ2-7	CASSWGYEQYF	1.94%	8	ND		1
NOD24-B8	TRBV1	TRBJ1-6	CTCSPPGQGNSPLYF	2.04%	7	ND		1
NOD24-B9	TRBV13-2	TRBJ2-5	CASGGDWGGQDTQYF	1.37%	9	ND		1
NOD24-B10	TRBV20	TRBJ1-2	CGARATGGSSDYTF	1.25%	11	ND		1
NOD24-B11	TRBV16	TRBJ1-1	CASSLDRNTEVFF	1.28%	10	ND		1
NOD24-B12	TRBV13-2	TRBJ2-7	CASGGYEQYF	1.07%	14	0.01%	667	3
NOD25-B13	TRBV13-1	TRBJ2-5	CASSDGTGEDTQYF	ND		11.67%	1	2
NOD25-B14	TRBV1	TRBJ2-2	CTCSPPGQNTGQLYF	ND		6.34%	2	1
NOD25-B15	TRBV13-1	TRBJ2-7	CASSYRGGEQYF	ND		5.09%	3	1
NOD25-B16	TRBV13-3	TRBJ2-4	CASSDSQNTLYF	0.22%	86	3.95%	4	5
NOD25-B17	TRBV1	TRBJ2-4	CTCSAELGGQNTLYF	ND		3.75%	5	1
NOD25-B18	TRBV13-3	TRBJ2-4	CASSADWENTLYF	ND		3.65%	6	1
NOD25-B19	TRBV20	TRBJ2-1	CGARVGGRYYAEQFF	ND		3.56%	7	1
NOD25-B20	TRBV13-3	TRBJ2-4	CASSDAQNTLYF	ND		1.58%	11	1
NOD25-B22	TRBV15	TRBJ1-2	CASSLGQGDSDYTF	ND		1.50%	12	1
NOD25-B23	TRBV1	TRBJ1-6	CTCSAGQGNSPLYF	ND		1.90%	8	1
NOD25-B24	TRBV5	TRBJ2-4	CASSQDFQSQNTLYF	ND		1.47%	13	1
NOD25-B25	TRBV31	TRBJ1-1	CAWSQRTGVGTEVFF	ND		1.62%	10	1
NOD25-B26	TRBV13-1	TRBJ1-4	CASSVDGDERLFF	ND		1.18%	14	1

ND: Not detected

Supplemental Table 5

Supplemental Table 5: TCR sequencing of NOD mouse islets and peripheral immune organs

Islets	Total alpha in-frame reads	Unique alpha in-frame clonotypes	Total beta in-frame reads	Unique beta in-frame clonotypes
NOD26	985,010	16,557	657,623	11,764
NOD28	1,057,459	24,533	1,119,029	28,282
NOD29	1,138,110	22,649	951,905	22,221
Pancreatic lymph nodes	Total alpha in-frame reads	Unique alpha in-frame clonotypes	Total beta in-frame reads	Unique beta in-frame clonotypes
NOD26	2,769,784	188,098	3,398,515	250,607
NOD28	2,275,840	294,930	4,323,129	722,413
NOD29	3,222,389	254,462	3,363,743	326,212
Blood	Total alpha in-frame reads	Unique alpha in-frame clonotypes	Total beta in-frame reads	Unique beta in-frame clonotypes
NOD26	2,099,445	107,545	3,260,187	203,538
NOD28	1,540,902	62,400	2,821,750	161,740
NOD29	1,744,235	97,847	3,442,126	241,490

Supplemental Table 6

Supplemental Table 6: Statistical analysis evaluating the correlation of TCR frequencies between the islets and PLN or blood

Rank (Alpha)	Estimated difference (PLN-Alpha – Blood-Alpha) ± standard error	P-Value
10	83.333 ± 5.282	<0.001
100	46.667 ± 5.282	0.001
1000	37.067 ± 5.282	0.002
all	6.757 ± 5.282	0.270
Rank (Beta)	Estimated difference (PLN-Beta – Blood-Beta) ± standard error	P-Value
10	70.000 ± 5.623	<0.001
100	58.000 ± 5.623	<0.001
1000	38.267 ± 5.623	0.002
all	8.267± 5.623	0.215

Supplemental Table 7

Supplemental Table 7: Mice used for the Longitudinal analysis of NY8.3 clonotype frequencies in the blood

Cohort*	Strain	Mouse ID	Age of diabetes (weeks)	Litter information	Sampling 1 weeks of age (Total in-frame reads)	Sampling 2 weeks of age (Total in-frame reads)	Sampling 3 weeks of age (Total in-frame reads)	Sampling 4 weeks of age (Total in-frame reads)
1	WT NOD	NOD104	18	A	4 (1,069,916)	10 (1,223,792)	15 (1,613,552)	18 (2,092,326)
1	WT NOD	NOD105	18	A	4 (1,711,121)	10 (2,175,181)	15 (2,615,307)	18 (1,991,711)
1	WT NOD	NOD106	No**	A	4 (1,558,062)	10 (1,333,804)	15 (2,135,266)	21 (1,649,784)
1	WT NOD	NOD107	17	B	5 (1,648,218)	10 (1,442,495)	15 (1,927,723)	17 (1,880,318)
1	WT NOD	NOD108	17	B	5 (2,123,119)	10 (2,930,264)	15 (1,535,585)	17 (2,362,644)
1	WT NOD	NOD110	12	B	5 (1,249,134)	10 (1,378,383)	12 (1,896,159)	NS***
1	WT NOD	NOD111	18	C	7 (1,078,530)	11 (1,292,589)	15 (1,378,802)	18 (2,138,628)
1	WT NOD	NOD112	16	D	5 (1,587,113)	11 (1,552,225)	15 (2,215,495)	16 (2,558,196)
1	WT NOD	NOD115	No**	D	5 (1,338,895)	11 (1,909,489)	15 (2,606,266)	22 (1,366,869)
1	Insulin-KO	FKO101	No**	E	5 (1,973,357)	10 (1,122,121)	15 (1,226,931)	20 (1,813,873)
1	Insulin-KO	FKO103	No**	F	5 (1,610,991)	10 (581,322)	14 (855,928)	20 (909,855)
1	Insulin-KO	FKO104	No**	F	5 (1,281,060)	10 (1,789,709)	14 (1,030,923)	20 (1,484,822)
1	Insulin-KO	FKO106	No**	G	5 (1,249,998)	11 (2,226,010)	15 (1,193,615)	20 (2,460,945)
1	Insulin-KO	FKO107	No**	G	5 (1,563,752)	11 (2,174,681)	15 (2,151,118)	20 (2,314,362)
1	Insulin-KO	FKO108	No**	G	5 (1,122,873)	11 (1,213,187)	15 (886,480)	20 (1,375,617)
1	Insulin-KO	FKO109	No**	H	5 (1,336,809)	11 (1,468,089)	15 (1,327,351)	20 (1,616,527)
2	WT NOD	C3	No**	I	5 (4,193,573)	9 (4,055,747)	14 (4,656,857)	19 (5,141,010)
2	WT NOD	C5	17	J	5 (10,576,883)	9 (13,777,653)	14 (13,296,573)	17 (15,480,063)
2	WT NOD	C6	16	J	5 (4,317,153)	9 (3,163,090)	14 (4,569,335)	16 (3,768,299)
2	WT NOD	C7	17	K	4 (12,436,138)	9 (13,251,127)	14 (17,796,141)	17 (12,149,154)
2	WT NOD	C8	No**	K	4 (5,303,064)	9 (4,717,092)	14 (3,600,914)	19 (4,929,517)
2	WT NOD	C11	No**	L	4 (2,314,130)	9 (3,387,880)	14 (3,226,386)	19 (2,993,437)
2	WT NOD	C12	17	L	4 (8,236,554)	9 (8,745,496)	14 (9,542,150)	17 (10,060,768)
2	WT NOD	C13	19	L	4 (7,710,946)	9 (5,404,204)	14 (4,744,058)	19 (7,547,577)
2	WT NOD	C14	No**	L	4 (9,340,808)	9 (4,937,777)	14 (4,851,409)	19 (4,719,803)
2	WT NOD	C15	13	M	5 (7,418,086)	10 (5,112,072)	13 (6,795,967)	NS***
2	WT NOD	C17	15	N	5 (7,042,369)	10 (4,591,012)	15 (5,595,926)	NS***
2	WT NOD	C18	16	N	5 (10,063,480)	10 (12,022,105)	15 (16,198,920)	16 (13,139,413)
2	Insulin-KO	KO1	No**	O	5 (8,923,585)	10 (7,273,109)	15 (6,216,387)	19 (8,129,167)
2	Insulin-KO	KO2	No**	O	5 (8,780,928)	10 (6,184,497)	15 (5,250,893)	19 (7,484,916)
2	Insulin-KO	KO3	No**	O	5 (8,052,439)	10 (6,391,635)	15 (7,100,899)	19 (7,383,013)
2	Insulin-KO	KO4	No**	O	5 (6,153,258)	10 (7,382,593)	15 (8,080,229)	19 (7,540,552)
2	Insulin-KO	KO5	No**	P	5 (3,511,083)	11 (4,149,135)	15 (4,690,297)	20 (3,174,493)
2	Insulin-KO	KO6	No**	Q	5 (4,690,744)	10 (5,489,943)	14 (3,609,444)	19 (3,146,172)
2	Insulin-KO	KO7	No**	Q	5 (5,309,687)	10 (3,980,164)	14 (4,612,929)	19 (3,234,381)
2	Insulin-KO	KO8	No**	Q	5 (4,727,883)	10 (2,824,690)	14 (3,563,029)	19 (4,137,345)

* Cohort 1: animals analyzed for the results shown in Figure 4 and Supplemental Figure 5.
Cohort 2: animals analyzed for the results shown in Supplemental Figure 6.

** non-diabetic

*** Not sampled; sacrificed at diabetes onset

Supplemental Table 8

Supplemental Table 8: Demographics of organ donors for islets and pancreas slice sampling

	T1D	AAb+	Non-diabetic
Number	14	4	9
Age (years): Mean (SD)	18.1 (9.0)	22.3 (4.6)	26.2 (6.3)
Age (years): Range	3-28	19-29	18-34
Gender: Female %	64%	50%	33%
HLA: DR4-DQ8 % present	50%	25%	11%
HLA: DR3-DQ2 % present	50%	50%	44%
HLA: A2 % present	57%	75%	56%
Number of CD4 T cells analyzed	2,580	355	474
Number of in-frame TCRs detected (CD4-alpha)	2,191	390	427
Number of in-frame TCRs detected (CD4-beta)	2,279	344	405
Number of unique TCR clonotypes identified (CD4-alpha)	1,843	323	343
Number of unique TCR clonotypes identified (CD4-beta)	1,923	281	326
Number of CD8 T cells analyzed	2,788	849	1,272
Number of in-frame TCRs detected (CD8-alpha)	2,543	933	1,053
Number of in-frame TCRs detected (CD8-beta)	2,420	827	1,073
Number of unique TCR clonotypes identified (CD8-alpha)	1,596	495	616
Number of unique TCR clonotypes identified (CD8-beta)	1,564	463	617

SD: standard deviation

DR4-DQ8: DRB1*04:0X-DQA1*03:01-DQB1:03:02

DR3-DQ2: DRB1*03:01-DQA1*05:01-DQB1:02:01

A2: A*02:01

Supplemental Table 9

Supplemental Table 8: TCR sequencing information of human islets and pancreas slice samples

				Number of in-frame TCRs detected			
Donor Type	DR4-DQ8	DR3-DQ2	A2	CD4_alpha	CD4_beta	CD8_alpha	CD8_beta
T1D	Yes	No	Yes	10	7	43	42
T1D	Yes	Yes	No	252	282	120	145
T1D	Yes	No	Yes	351	428	173	182
T1D	Yes	No	Yes	166	157	170	183
T1D	No	Yes	No	224	210	106	121
T1D	No	No	No	25	35	138	193
T1D	Yes	Yes	Yes	76	101	201	217
T1D	No	No	No	74	142	48	76
T1D	No	Yes	No	65	59	192	178
T1D	Yes	Yes	Yes	154	145	443	343
T1D	Yes	Yes	Yes	243	213	229	180
T1D	No	Yes	No	263	251	190	198
T1D	No	No	Yes	97	83	314	229
T1D	No	No	Yes	191	166	176	133
AAb+	No	No	Yes	269	241	385	358
AAb+	No	Yes	Yes	66	56	350	290
AAb+	No	Yes	Yes	20	13	53	50
AAb+	Yes	No	No	35	34	145	129
Non-diabetic	No	Yes	Yes	52	49	152	155
Non-diabetic	No	Yes	No	56	40	143	152
Non-diabetic	No	No	Yes	13	17	52	54
Non-diabetic	No	No	Yes	13	24	163	172
Non-diabetic	No	No	Yes	0	0	71	74
Non-diabetic	No	No	No	0	0	62	55
Non-diabetic	No	Yes	No	19	14	199	204
Non-diabetic	No	Yes	No	174	159	103	98
Non-diabetic	Yes	No	Yes	100	102	108	109

DR4-DQ8: DRB1*04:0X-DQA1*03:01-DQB1:03:02

DR3-DQ2: DRB1*03:01-DQA1*05:01-DQB1:02:01

A2: A*02:01

Supplemental Table 10

Supplemental Table 10: Unique TCR clonotypes detected in the pancreas of two or more donors

T cell subset	Chain Type	V-gene	J-gene	Junction	Donors (number)			HLA alleles shared among donors	Known specificity to preproinsulin
					T1D	AAb+	Non-diabetic		
CD4	Alpha	TRAV8-1	TRAJ26	CAVNGDNYGQNFVF	2	0	0	DRB1*04:0X, DQA1*03:01, DQB1*03:02, DPA1*01:03, DPB1*04:01	
CD4	Alpha	TRAV36/DV7	TRAJ44	CAVETGTASKLTF	2	0	0	DRB1*04:0X, DQA1*03:01, DQB1*03:02, DPA1*01:03	
CD4	Alpha	TRAV4	TRAJ8	CLVGDMNTGFQKLVF	2	0	0	DRB1*03:01, DQA1*05:01, DQB1*02:01, DPA1*01:03	
CD4	Alpha	TRAV13-1	TRAJ17	CAASIKAAGNKLTF	2	0	0	DPA1*01:03, DPB1*04:01	
CD4	Alpha	TRAV14/DV4	TRAJ54	CAMREGVQGAQKLVF	2	0	0	DPA1*01:03	
CD4	Alpha	TRAV8-2/8-4	TRAJ17	CAVSKAAGNKLTF	1	1	0	DPA1*01:03, DPB1*04:01	
CD4	Alpha	TRAV13-1	TRAJ40	CAASRTTSGTYKYIF	1	0	1	DRB1*04:04, DQA1*03:01, DQB1*03:02, DPA1*01:03, DPA1*02:01	
CD4	Alpha	TRAV13-1	TRAJ53	CAASGGSNYKLTF	1	0	1	DPA1*01:03, DPB1*04:01	
CD4	Alpha	TRAV16	TRAJ27	CALNTNAGKSTF	1	0	1	DPA1*01:03, DPB1*04:01	
CD4	Alpha	TRAV29/DV5	TRAJ42	CAATFYGGSQGNLIF	0	0	2	DQA1*03:03, DQB1*03:02, DPA1*01:03	
CD4	Beta	TRBV20-1	TRBJ1-3	CSARGPNSGNTIYF	2	0	0	DPA1*01:03, DPB1*04:01	
CD4	Beta	TRBV27	TRBJ2-3	CASSLWTSSDTQYF	1	0	1	DRB1*04:04, DQA1*03:01, DQB1*03:02, DPA1*01:03, DPA1*02:01	
CD8	Alpha	TRAV19	TRAJ49	CALSEAHTGNQFYF	2	0	0	A*02:01, B*40:01, C*03:04	
**CD8**	**Alpha**	**TRAV3**	**TRAJ26**	**CAVPDNYGQNFVF**	**2**	**0**	**0**	**A*01:01, B*08:01, C*07:01**	**PPI:96-103**
CD8	Alpha	TRAV3	TRAJ37	CAVPSNTGKLIF	2	0	0	A*01:01, B*08:01, C*07:01	
**CD8**	**Alpha**	**TRAV19**	**TRAJ9**	**CALTLNTGGFKTIF**	**2**	**0**	**0**	**B*08:01, C*07:01**	**PPI:33-41**
CD8	Alpha	TRAV8-1	TRAJ39	CAVNNNAGNMLTF	2	0	0	B*08:01, C*07:01	
**CD8**	**Alpha**	**TRAV8-1**	**TRAJ13**	**CAVNAAGGYQKVTF**	**2**	**0**	**0**	**None**	**PPI:1-11**
CD8	Alpha	TRAV19	TRAJ42	CALSEAGNYGGSQGNLIF	2	1	0	A*02:01	
CD8	Alpha	TRAV21	TRAJ11	CAVNSGYSTLTF	1	1	0	A*01:01, B*08:01, B*07:02, C*07:01, C*07:02	
**CD8**	**Alpha**	**TRAV29/DV5**	**TRAJ57**	**CAASAGGGSEKLVF**	**1**	**1**	**0**	**A*01:01, A*02:01, B*08:01, C*07:01**	**PPI:2-11**
CD8	Alpha	TRAV13-1	TRAJ45	CAASISGGGADGLTF	1	1	0	A*02:01, C*07:01	
CD8	Alpha	TRAV19	TRAJ35	CALSGFGNVLHC	1	1	0	A*02:01	
CD8	Alpha	TRAV12-2	TRAJ7	CAVRYGNNRLAF	1	1	1	A*01:01, B*08:01, C*05:01, C*07:01	
CD8	Alpha	TRAV26-2	TRAJ39	CILSKNAGNMLTF	1	0	1	A*02:01, B*40:01, C*03:04, C*07:0X	
CD8	Alpha	TRAV27	TRAJ42	CAGAGGGSQGNLIF	1	0	1	A*02:01, C*07:0X	
CD8	Alpha	TRAV12-2	TRAJ15	CAVNQAGTALIF	1	0	1	None	
CD8	Alpha	TRAV13-1	TRAJ26	CAANNYGQNFVF	0	1	1	A*02:01, A*30:02, C*07:0X	
CD8	Alpha	TRAV1-2	TRAJ4	CAGSGGYNKLIF	0	0	2	A*30:02, B*18:01, C*05:01, C*07:0X	
CD8	Beta	TRBV13	TRBJ2-3	CASSFGTDTQYF	2	0	0	C*03:04, C*07:0X	
CD8	Beta	TRBV19	TRBJ2-7	CASSIRSSYEQYF	2	0	1	A*02:01, C*07:01	
CD8	Beta	TRBV19	TRBJ2-3	CASSIRSTDTQYF	1	1	0	A*02:01, C*07:01	
CD8	Beta	TRBV7-8	TRBJ1-5	CASSLERGGGQPQHF	1	1	0	A*02:01, C*06:02	

CD8	Beta	TRBV9	TRBJ2-7	CASSVGQGTYEQYF	0	2	0	A*02:01	
CD8	Beta	TRBV7-9	TRBJ1-5	CASSLGQGDQPQHF	1	0	1	A*02:01	
CD8	Beta	TRBV20-1	TRBJ2-7	CSASSGYEQYF	0	0	2	A*30:02, B*18:01, C*05:01, C*07:0X	
CD8	Beta	TRBV27	TRBJ2-7	CASRGVSYEQYF	0	0	2	A*30:02, C*07:0X	

X: any allele number shared within a designated allele group

Supplemental Table 11

Supplemental Table 11: Boostrapping analysis controlling for differences in cell number comparing shared clonotype frequencies between CD4 and CD8-derived TCR clonotypes

	CD4 Alpha count	CD8 Alpha Mean count	CD8 Alpha SD	Alpha p value*
T1D	5	5.393	1.08983427	0.718
AAb+	0	0	0	not applicable
Non-diabetic	1	0.34	0.4739458	0.164
Between groups	4	4.617	1.44651423	0.67
	CD4 Beta count	CD8 Beta Mean count	CD8 Beta SD	Beta p value*
T1D	1	1.758	0.45567932	0.096
AAb+	0	0.425	0.49459036	0.39
Non-diabetic	0	0.646	0.65046684	0.321
Between groups	1	2.129	0.85503806	0.187

*P value calculated by Z-score

Supplemental Table 12

Supplemental Table 12: Clusters with or without preproinsulin-specific TCR clonotypes

	**Clusters containing preproinsulin-specific clonotypes**		**Clusters not containing preproinsulin-specific clonotypes**	
	**T1D or AAb**	**With Non-diabetic**	**T1D or AAb**	**With Non-diabetic**
CD4-Alpha	2	0	102	33
CD4-Beta	0	1	20	6
CD8-Alpha	12	2	85	68
CD8-Beta	5	0	27	29
**Total**	**19**	**3**	**234**	**136**
